# Operando high-pressure investigation of size-controlled CuZn catalysts for the methanol synthesis reaction

**DOI:** 10.1038/s41467-021-21604-7

**Published:** 2021-03-04

**Authors:** Núria J. Divins, David Kordus, Janis Timoshenko, Ilya Sinev, Ioannis Zegkinoglou, Arno Bergmann, See Wee Chee, Simon Widrinna, Osman Karslıoğlu, Hemma Mistry, Mauricio Lopez Luna, Jian Qiang Zhong, Adam S. Hoffman, Alexey Boubnov, J. Anibal Boscoboinik, Marc Heggen, Rafal E. Dunin-Borkowski, Simon R. Bare, Beatriz Roldan Cuenya

**Affiliations:** 1 Department of Physics, Ruhr University Bochum, Bochum, Germany; 2 Department of Interface Science, Fritz-Haber Institute of the Max Planck Society, Berlin, Germany; 3 Stanford Synchrotron Radiation Lightsource, SLAC National Accelerator Laboratory, Menlo Park, CA USA; 4 Center for Functional Nanomaterials, Brookhaven National Laboratory, Upton, New York, USA; 5 Ernst Ruska-Centre for Microscopy and Spectroscopy with Electrons and Peter Grünberg Institute, Forschungszentrum Jülich, Jülich, Germany; 6 Present Address: Institute of Energy Technologies, Universitat Politècnica de Catalunya, EEBE, Barcelona, Spain

**Keywords:** Catalytic mechanisms, Heterogeneous catalysis, Porous materials

## Abstract

Although Cu/ZnO-based catalysts have been long used for the hydrogenation of CO_2_ to methanol, open questions still remain regarding the role and the dynamic nature of the active sites formed at the metal-oxide interface. Here, we apply high-pressure operando spectroscopy methods to well-defined Cu and Cu_0.7_Zn_0.3_ nanoparticles supported on ZnO/Al_2_O_3_, γ-Al_2_O_3_ and SiO_2_ to correlate their structure, composition and catalytic performance. We obtain similar activity and methanol selectivity for Cu/ZnO/Al_2_O_3_ and CuZn/SiO_2_, but the methanol yield decreases with time on stream for the latter sample. Operando X-ray absorption spectroscopy data reveal the formation of reduced Zn species coexisting with ZnO on CuZn/SiO_2_. Near-ambient pressure X-ray photoelectron spectroscopy shows Zn surface segregation and the formation of a ZnO-rich shell on CuZn/SiO_2_. In this work we demonstrate the beneficial effect of Zn, even in diluted form, and highlight the influence of the oxide support and the Cu-Zn interface in the reactivity.

## Abstract (web summary)

The nature of the active species over Cu/ZnO catalysts for methanol synthesis remains elusive. Here, the authors shed light on the evolution of the nanoparticle/support interface and correlate its structural and chemical transformations with changes in the catalytic performance.

## Introduction

The utilization of fossil fuels as the main energy source gives rise to serious environmental issues, including global warming caused by the continuously increasing level of atmospheric CO_2_. The hydrogenation of CO_2_ is an important process to valorize CO_2_ by converting it into useful chemicals and fuels, such as methanol^1–4^. Methanol is presently industrially synthesized from CO_2_, CO, and H_2_ at pressures between 50 and 100 bar and temperatures between 200 and 300 °C using Cu/ZnO/Al_2_O_3_ catalysts^5–7^.

Although Cu/ZnO-based catalysts have been long studied, the role and the dynamic nature of the active sites that originate at the metal-oxide interface under the strongly reducing reaction conditions are still under debate. It has been shown that Cu-based catalysts containing ZnO are more active than their ZnO-free counterparts^8^ and that ZnO both enhances the dispersion of the Cu nanoparticles (NPs), and acts as an electronic promoter^8,9^ through a strong metal-support interaction^8–10^. This promoting effect of Zn has been attributed to the Zn being present in a number of different configurations: the formation of graphitic ZnO_x_ layers on Cu NPs^11^, metallic Zn atoms within the Cu NP surface^12^, CuZn alloy formation^13^, Zn-decoration of stepped Cu surfaces^1^, or the formation of ZnO at the interface with Cu in single crystals^3,14^. A strong correlation between the Zn coverage on Cu NPs and the methanol synthesis activity was also reported^2^. Furthermore, the structure-sensitivity and size-dependent activity for this reaction were shown for Cu and CuZn-based catalysts^4^.

Heterogeneous catalysts are dynamic entities and adapt their morphology and electronic structure to the chemical potential of the surrounding gaseous atmosphere^15,16^. Thus, for methanol synthesis catalysts, operando studies in pressure regimes that are industrially relevant (20–100 bar), as the ones discussed in the present work, are key to overcome the “pressure gap”^15,17–25^, as they probe Cu and Zn under their actual working state, unraveling their active chemical and structural state. Investigations on Cu/ZnO systems revealed the presence of poorly crystalline ZnO interacting with Cu^15^, reversible changes of the Cu NP morphology depending on the reaction conditions^17,23^, CuZn bulk alloy formation under severe reduction conditions (600 °C in CO/H_2_)^17,26^, and ZnO reduction and a stronger CuZn interaction for catalysts supported on SiO_2_^19–21^. Interestingly, even at sub-atmospheric pressures (1–2 mbar)^27^, the dynamic behavior of these catalysts has also been corroborated with the observation of wetting/non-wetting of Cu NPs on ZnO^28^. Despite the tremendous effort dedicated to the investigation of this reaction, questions still remain on whether metallic Zn/brass is present under industrially relevant reaction conditions, its role in the reaction selectivity, and whether ZnO is needed as a part of the support, or small ZnO ensembles in direct contact with Cu within a NP can also serve to activate CO_2_.

To address these questions, here we use morphologically and chemically well-defined Cu and CuZn NP catalysts that help close the materials gap between the heterogeneous industrial catalysts and the model single-crystal systems previously studied, while also addressing the pressure gap through operando spectroscopic characterization at industrially relevant high-pressure conditions. The small NP size chosen in our study allows us to investigate the changes occurring at the surface and near-surface region of the NPs by the bulk-sensitive technique X-ray absorption spectroscopy (XAS), since the fraction of atoms at the surface of Cu NPs of ca. 3 nm is ~60%^29^. In this way, we are able to shed light on the dynamic evolution of the metal NP/oxide support interface during CO_2_ + CO hydrogenation.

## Results

### Model catalysts synthesis and characterization

Five catalysts, designed to be analogous to the commercial Cu/ZnO/Al_2_O_3_ catalyst, composed of size-controlled NPs were synthesized to investigate the different components and interactions of the industrial catalyst. Monometallic Cu NPs were deposited on (i) ZnO/Al_2_O_3_, (ii) SiO_2_, (iii) γ-Al_2_O_3_, and bimetallic Cu_0.7_Zn_0.3_ NPs on (iv) SiO_2_, and (v) γ-Al_2_O_3_.

**Fig. 1:**
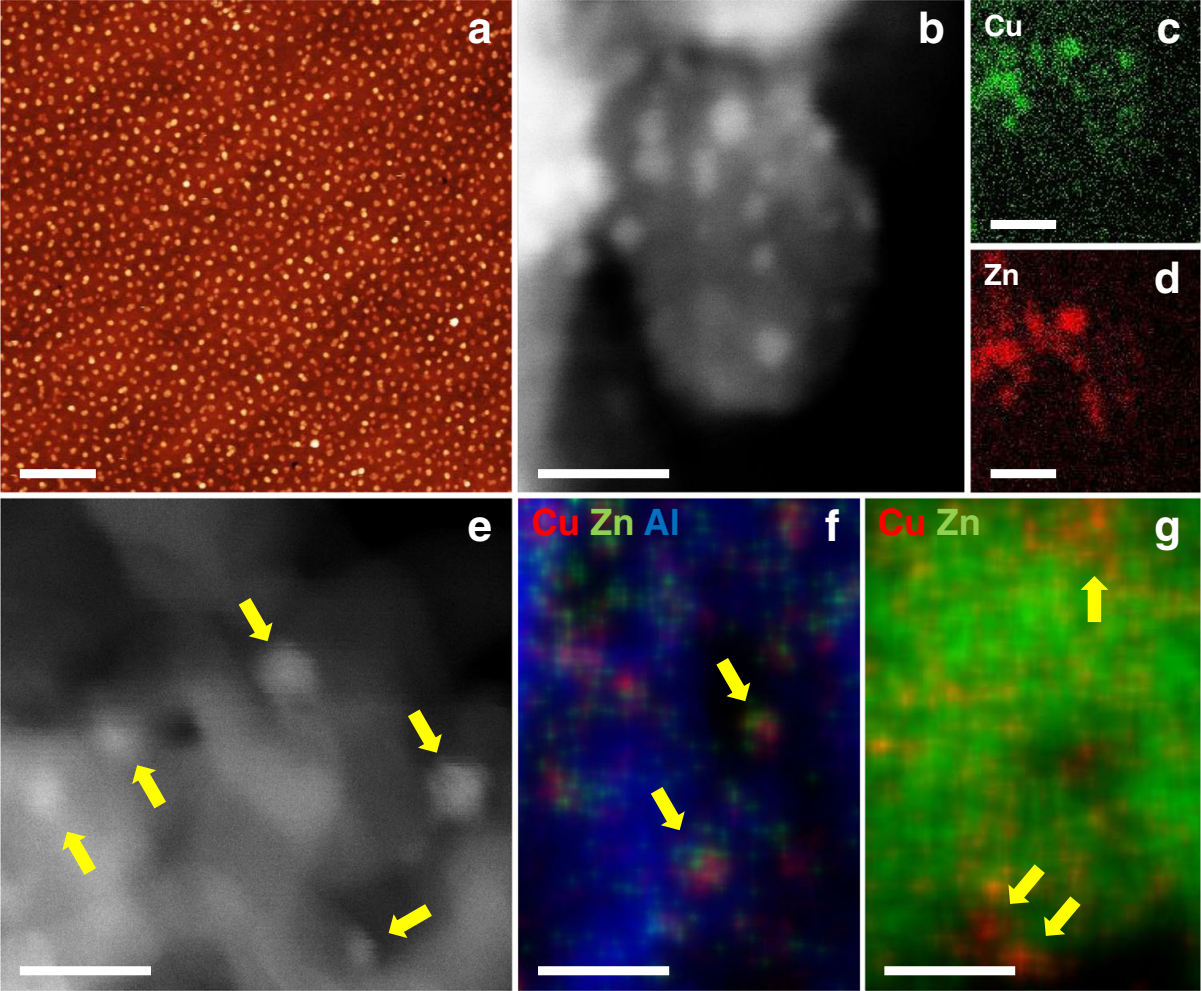
Microscopy characterization of the catalysts. **a** AFM image of the Cu NPs supported on SiO_2_/Si(111). **b** STEM image of the CuZn/SiO_2_ catalyst; **c**, **d** show the corresponding Cu and Zn EDX maps. **e** STEM image and **f** EDX map of the CuZn/Al_2_O_3_ catalyst. **g** EDX map of the Cu/ZnO/Al_2_O_3_ catalyst. In EDX maps **f**, **g** red represents Cu, green Zn and blue Al. The scale bars correspond to **a** 400 nm, **b**–**g** 10 nm.

The NPs were prepared following an inverse micelle encapsulation method. Figure 1a shows an atomic force microscopy (AFM) image of the Cu NPs with a mean height of 3.1 ± 0.8 nm. Similar data were obtained for the Cu_0.7_Zn_0.3_ NPs (Supplementary Figure 1). A Cu/Zn ratio of 70/30 was chosen for the bimetallic NPs to assure sufficient promotion of Cu by Zn^4^. The powder catalysts were synthesized by incipient wetness impregnation of the pre-formed NP solutions on the oxide supports (Supplementary Figure 2) and calcined at temperatures ≥400 °C for 6 h in 20% O_2_/(Ar or N_2_) to completely remove the polymer. Figure 1b shows a scanning transmission electron microscopy (STEM) image of the CuZn/SiO_2_ catalyst. The mean particle size determined by TEM after calcination is 2.7 ± 0.7 nm. Energy-dispersive X-ray spectroscopy (EDX) maps in Fig. 1c and d confirm that the Cu and Zn signals, respectively, originate from the same NP. Figure 1e displays a STEM image of the CuZn/Al_2_O_3_ sample showing isolated NPs. Figure 1f shows an EDX map overlayed with a high-angle annular dark-field image. The mean NP diameter determined by TEM is 3.3 ± 0.7 nm. In Fig. 1g an EDX map for the Cu/ZnO/Al_2_O_3_ catalyst is displayed, showing Cu distributed over the ZnO/Al_2_O_3_ support. Additional TEM images are shown in Supplementary Figures 3–5.

X-ray diffraction (XRD) patterns of the Cu/ZnO/Al_2_O_3_, CuZn/SiO_2_, and CuZn/Al_2_O_3_ catalysts were recorded in their calcined state and after reaction. A detailed description of the findings is included in the Supplementary Discussion, and Supplementary Figure 6. Note here that XRD is not sensitive to small atomically disordered NPs, and therefore, the results obtained are biased to the minority fraction of agglomerated NPs in these samples.

### Catalyst structural evolution under a high-pressure environment

To gain insight into the interaction between Cu and Zn and into the evolution of their chemical state and structure under methanol synthesis conditions, the Cu/ZnO/Al_2_O_3_, CuZn/Al_2_O_3_, and CuZn/SiO_2_ catalysts were investigated by means of operando XAS at the Cu and Zn K-edges. The study of nano-sized pre-formed CuZn NPs allowed us to clearly track the evolution of the Zn species. This would be challenging to implement for the industrial catalyst as it contains Zn as the bulk support.

XAS data of the fresh calcined samples were acquired under He at room temperature, followed by a reduction (activation) under 10% H_2_/He for 2 h at 245 °C for the Cu/ZnO/Al_2_O_3_ and the CuZn/Al_2_O_3_ catalysts and at 325 °C for the CuZn/SiO_2_ catalyst^19^. A higher reduction temperature was used for the NPs deposited on SiO_2_ owing to the higher stability of the CuO_x_ species on this support. Further details on the effect of the reduction temperature are given in the Supplementary Discussion. Next, the samples were studied under an industrially relevant methanol synthesis mixture containing 4% CO_2_, 10% CO, 72% H_2_, balanced in He, at: (i) 20 bar at 220 °C, (ii) 20 bar at 280 °C, (iii) 40 bar at 280 °C, and (iv) 40 bar at 320 °C. The last step at 320 °C was performed in order to mimic an ageing treatment.

**Fig. 2:**
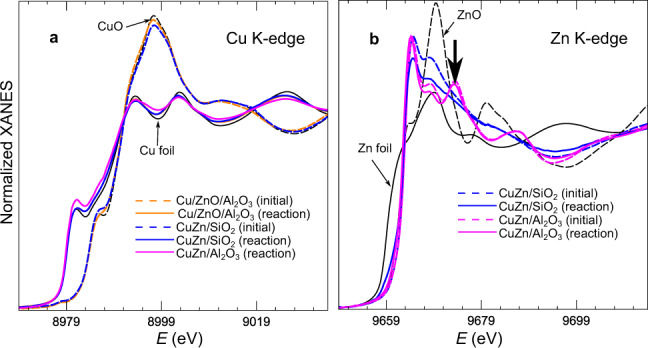
Operando XANES spectra. **a** Cu K-edge and **b** Zn K-edge XANES spectra for Cu/ZnO/Al_2_O_3_, CuZn/SiO_2_ and CuZn/Al_2_O_3_ catalysts in their initial state (dashed lines), and under operando conditions in CO_2_+CO+H_2_, *p* = 20 bar, *T* = 220 °C (solid lines). The reference spectra for CuO and ZnO (dashed lines), Cu and Zn foils (solid lines) are also shown. The vertical arrow in **b** marks the signature of the zinc spinel structure.

**Fig. 3:**
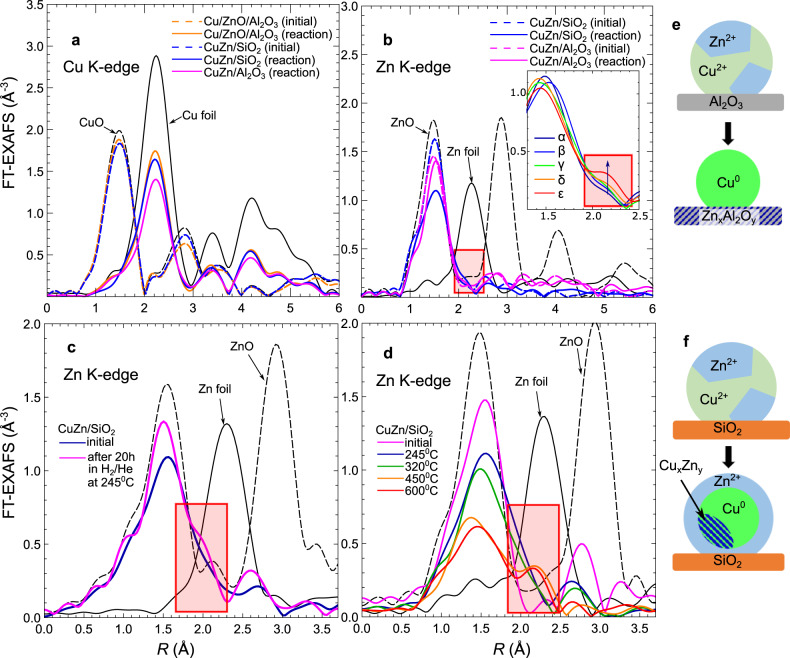
Operando Cu K-edge and Zn K-edge EXAFS spectra. The FT magnitude of the **a** Cu K-edge and **b** Zn K-edge EXAFS spectra for Cu/ZnO/Al_2_O_3_, CuZn/SiO_2_, and CuZn/Al_2_O_3_ catalysts in their initial state (dashed lines), and under operando conditions in CO_2_+CO+H_2_, *p* = 20 bar, *T* = 220°C (solid lines). The magnitude of the FT of the EXAFS spectra for CuZn/SiO_2_ during **c** a reduction in 10% H_2_/He for 20 h at 245°C and **d** at temperatures between 245 and 600°C. The reference spectra for a Zn foil, ZnO, CuO, and Cu foil are also shown. The highlighted areas in **b**–**d** indicate the region where the Zn–M bond develops under reaction conditions. The latter is also shown in the inset in **b**, with spectra *α*, *β*, *γ*, *δ,* and *ε* corresponding to consequent measurements at 220°C/20 bar, 280°C/20 bar, 280°C/40 bar, and 320°C/40 bar. To enhance the display of the referred features, different k-ranges were used for the Fourier transform in **a**, **b** 1–10.5 Å^−1^, **c** 3–12 Å^−1^, and **d** 1–10 Å^−1^. The observed changes are schematically presented in **e** for CuZn/Al_2_O_3_ and **f** for CuZn/SiO_2_.

Figure 2 shows Cu K-edge and Zn K-edge X-ray absorption near-edge structure (XANES) data and Fig. 3 shows the magnitude of the Fourier transform (FT) of the extended X-ray absorption fine-structure (EXAFS) data of the three catalysts measured in their initial state and under the methanol synthesis mixture at 20 bar and 220 °C (XAS data for the other reaction regimes are shown in Supplementary Figures 9–16). At the Cu K-edge, we observe that Cu species are initially oxidized and exhibit a local structure similar to CuO. Upon reduction in hydrogen and under reaction conditions, these species are reduced and remain reduced (Cu^0^) (see Figs. 2a and 3a, Supplementary Figures 10 and 11 and Supplementary Table 8). EXAFS data (see Supplementary Discussion) indicate that, although some NP sintering was revealed by XRD and TEM (Supplementary Figures 3–6), the majority of Cu is available within well-dispersed NPs that remain stable during the reaction. This follows from the observation that the 1^st^ shell coordination numbers (CNs) for reduced Cu are ca. 8–10, in agreement with NP sizes of ca. 3 nm^30^. Importantly, no changes in the CNs were observed under the different reaction conditions, suggesting that the majority of particles retained their size, shape, and structure.

Notably, the Zn K-edge XAS data are substantially different for CuZn/SiO_2_ and CuZn/Al_2_O_3_, revealing the distinct interaction between the CuZn NPs and the silica and alumina supports. The Zn K-edge XANES spectra of CuZn/SiO_2_ and CuZn/Al_2_O_3_ catalysts in their initial state can be aligned well with the spectrum of wurtzite ZnO (where Zn is in 2+ state and tetrahedrally coordinated), two main features appearing at 9664 eV and 9669 eV in the CuZn/SiO_2_ catalyst. Nevertheless, the relative intensities of these features are different for CuZn/SiO_2_ and bulk ZnO, and in conjunction with the weak contribution of distant coordination shells in the EXAFS data, indicate that Zn is present in a disordered oxide phase^31^. For the CuZn/Al_2_O_3_ sample, the small peak in the Fourier-transformed Zn K-edge EXAFS spectra between 2.5 and 3.0 Å is more pronounced and the Zn K-edge XANES spectra have an additional feature at ca. 9674 eV (see arrow in Fig. 2b). Similar XANES fingerprints were previously observed for various spinel structures such as ZnAl_2_O_4_^32^, indicating the migration of Zn atoms into the alumina support.

Under operando conditions, only minor changes can be observed for the Zn K-edge XAS of CuZn/Al_2_O_3_, suggesting the lack of significant reduction of the Zn species, which already incorporated in the Al_2_O_3_ matrix during the calcination. More pronounced changes are observed for the CuZn/SiO_2_: a decrease of the intensity of the main Zn K-edge XANES features and the appearance of a shoulder at the edge onset, characteristic for metallic Zn (Fig. 2b and Supplementary Figure 14). These changes can be related to the partial reduction of Zn species in this sample, and can be more easily observed in the Zn K-edge EXAFS spectra (Fig. 3b and Supplementary Figure 16), where an additional feature appears at ca. 2.2 Å in the FT and systematically increases under reaction conditions. This feature can be linked to the formation of Zn–Zn or Zn–Cu bonds and its intensity is low after a 2 h reduction at 325 °C (or during the first measurements under operando conditions), but becomes more pronounced with increasing reaction time. To confirm that the development of this feature was related to the prolonged exposure to reducing conditions, we investigated the effect of reducing atmospheres at different temperatures on the reduction of Zn. We collected the Zn K-edge XAS data for CuZn/SiO_2_ after 20 h under 10% H_2_/He at 245 °C and 1 bar (Fig. 3c and Supplementary Figure 17), and observed similar changes: the peak at ca. 2.2 Å related to Zn–M bonds clearly develops. This long-term study thus shows an increasing metallic Zn contribution for the CuZn/SiO_2_ NPs after the 20 h, as compared with that after 1–2 h of the reduction (Fig. 3d), indicating slowly progressing reduction of Zn species. To further corroborate this finding, we performed an additional experiment where the CuZn/SiO_2_ sample was investigated under 10% H_2_/He at 1 bar and temperatures from 245 °C up to 600 °C (Fig. 3d and Supplementary Figure 17). EXAFS data fitting (Supplementary Figures 12, 13, and 18, and Supplementary Table 9) confirmed the gradual transformation of initially tetrahedrally coordinated oxidized Zn species into a material with coexisting ZnO and reduced Zn species.

### Surface reorganization and oxidation state evolution

**Fig. 4:**
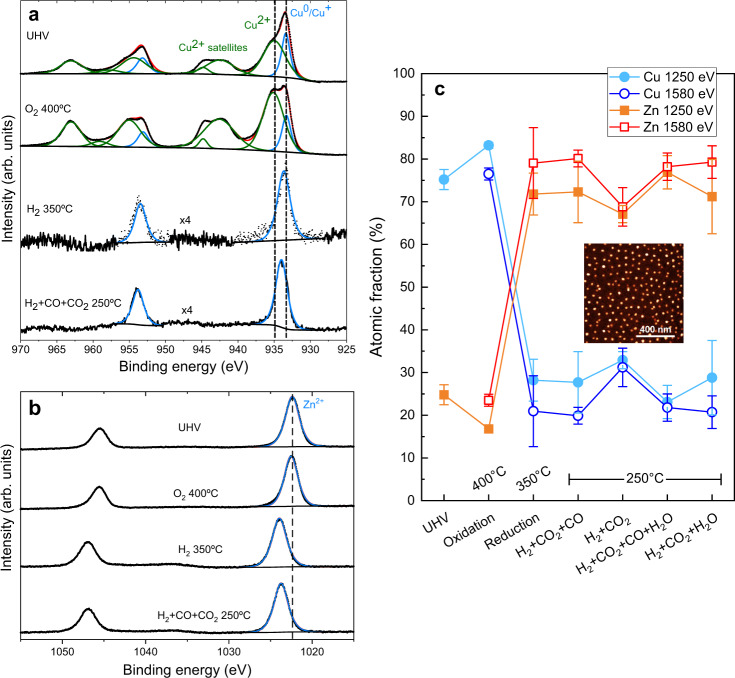
Near-ambient pressure X-ray photoelectron spectra of CuZn NPs on SiO_2_/Si(100). **a** Cu *2p* and **b** Zn *2p* regions recorded using a photon energy of 1250 eV. **c** Copper and zinc atomic fractions obtained under different atmospheres as indicated on the plot. The two-photon energies used for probing the surface (1250 eV) and subsurface (1580 eV) regions of the CuZn NPs are displayed. Error bars correspond to the standard deviation. The inset shows an AFM image of the sample.

To complement the bulk chemical and structural information obtained by XAS and gain further insight on the outermost layers of the NPs, synchrotron near-ambient pressure X-ray photoelectron spectroscopy (NAP-XPS) measurements were carried out. With this method, further knowledge was attained on the evolution of the oxidation state and surface composition of 6 nm Cu_0.7_Zn_0.3_ NPs deposited on SiO_2_/Si(100) (Fig. 4 and Supplementary Figure 19). In particular, depth profile information of the NP composition was obtained by choosing two different incident photon energies: 1250 eV, corresponding to inelastic mean free paths (IMFP) of ~0.8 nm for Cu *2p* and 0.7 nm for Zn *2p* photoelectrons, and 1580 eV corresponding to ~1.2 nm IMFP for Cu *2p* and Zn *2p*^33^. The series of in situ NAP-XPS data consisted of: (i) oxidation at 400 °C in 0.13 mbar O_2_ to remove adventitious carbon, (ii) reduction at 350 °C in 1.3 mbar H_2_, and (iii) reaction at 250 °C in 1.3 mbar of the reaction gas mixture. The effects of different reaction mixtures (CO_2_+CO+H_2_ versus CO_2_+H_2_) and the presence of water^34^ on the surface reorganization and chemical state of different species present during the reaction were studied (see concentrations in Supplementary Table 7).

The series of Cu *2p*_3/2_ and Zn *2p*_3/2_ NAP-XPS spectra, as well as the X-ray generated Cu and Zn LMM Auger regions are shown in Figs. 4a, b and Supplementary Figures 20–23. In the initial state under ultra-high vacuum (UHV) conditions, the Cu and Zn atomic ratios are 75:25, close to the nominal 70:30 synthesis ratio. Cu *2p* shows the typical satellites of Cu^2+^^35^, but can only be adequately fitted with an additional component of Cu^+^ or Cu^0^ (ca. 25%); Zn *2p* indicates the presence of ZnO. During the initial treatment in O_2_ at 400 °C, copper gets almost fully oxidized and the Cu/Zn ratio at the surface increases slightly, indicating a slight Cu surface segregation (Fig. 4c). The deconvolution of the Zn Auger region shows that zinc is present as ZnO (Supplementary Figure 22). Upon exposure to H_2_ at 350 °C, strong segregation of Zn toward the surface and of Cu toward the core of the NPs is observed, indicating the formation of a Zn-rich overlayer on the NPs. The Zn atomic content at the surface reached ~72%. This is in agreement with previous reports^1,36^ and responds to the higher stability of ZnO as compared with CuO in a reductive atmosphere. A shift toward higher binding energies (BEs) of the Zn *2p* and Cu *2p* and the Auger regions was observed, which can be due to charging effects of the SiO_2_ substrate. Under H_2_ atmosphere, copper gets fully reduced, which can be confirmed by the Cu LMM Auger spectrum (Supplementary Figure 23)^35^. At the same time, the shift of the Zn *2p* region towards higher BEs indicates that Zn mainly remains in Zn^2+^ state, either in the form of ZnO or Zn(OH)_2_. Analysis of the Zn LMM region, which is more sensitive to the Zn oxidation state, suggests a maximum of 5% metallic Zn coexisting with ZnO. It has been previously reported that the ZnO overlayer formed corresponds to distorted ZnO^11,37^, which is also in good agreement with our in situ XAS results.

Under reaction conditions at 250 °C with the H_2_+CO+CO_2_ mixture (as used before), no further reorganization of the metals is observed. Cu remains reduced and the Zn LMM lines seem to indicate a slight increase (up to 9%) in the concentration of metallic Zn. This indicates that under methanol synthesis conditions, the surface of the NPs remains Zn-rich, with a distorted ZnO_x_ layer located on top of the Cu NPs. Interestingly, this applies to the water-containing mixtures as well. Only under H_2_+CO_2_, a slight Cu surface segregation is observed, reaching 33 at.% Cu at the surface.

To bridge the pressure gap and display the relevance of these surface-sensitive experiments for the high-pressure regime, quasi in situ XPS measurements in combination with a high-pressure reactor cell (HPC) were carried out (Supplementary Figure 25). For this purpose, the same experimental conditions as for the synchrotron-based operando NAP-XPS measurements were applied in the HPC, but at more relevant pressures (1 bar for the reduction and 20 bar in the reaction mixture), before measuring the sample with XPS in UHV. A detailed description of these experiments can be found in the Supplementary Discussion. It is shown that there is no pressure effect for the segregation trends of these samples and therefore, our NAP-XPS synchrotron results stay valid even at higher pressures.

### Catalytic performance

**Fig. 5:**
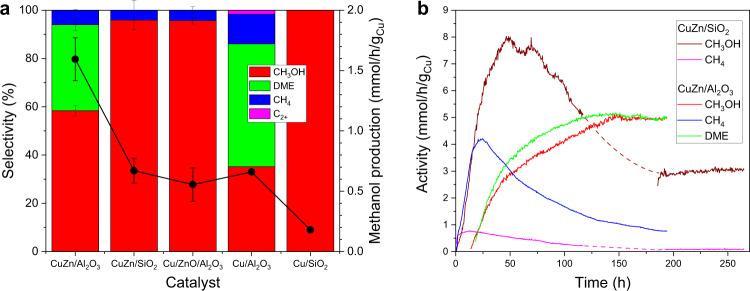
Catalytic performance and evolution of the reaction products during the activation. **a** Comparison of the methanol production and overall selectivity of all catalysts at 250°C and 40 bar. The C_2+_ products are mainly C_2_H_6_. Error bars represent standard deviation. **b** Temporal evolution of the main reaction products of the CuZn/SiO_2_ and CuZn/Al_2_O_3_ catalysts at 40 bar and 280°C during an activation treatment until the steady state was achieved. The dashed lines serve as a guide for the eye.

The activity of the catalysts in powder form was tested in a fixed-bed reactor at 20, 40, and 60 bar, at temperatures from 220 °C to 280 °C (later described as “reaction cycle”) in a reactant mixture of CO_2_/CO/H_2_/He=4/10/74/12, with a weight-to-flow ratio W/F = 0.18 g s/mL. Figure 5a and Supplementary Figure 7 display the steady-state reactivity data of our catalysts at different temperatures and pressures after an activation pre-treatment.

As shown in Fig. 5a, under steady-state operation and regardless of the support, the bimetallic CuZn NPs produce always more methanol than their monometallic Cu counterparts. The highest methanol yield was obtained for CuZn/Al_2_O_3_, followed by CuZn/SiO_2_, Cu/ZnO/Al_2_O_3_, and Cu/Al_2_O_3_, and a lower rate was observed for Cu/SiO_2_. Interestingly, CuZn/SiO_2_ is significantly less active than CuZn/Al_2_O_3_, but comparable in activity to Cu/ZnO/Al_2_O_3_. In addition, the stabilization of the methanol production for the CuZn/SiO_2_ catalyst took longer, and a significant deactivation after an initial increase of the methanol production was observed. The temporal evolution of the methanol production of the CuZn/SiO_2_ and CuZn/Al_2_O_3_ samples (at 40 bar and 280 °C) for 250 h is displayed in Fig. 5b, featuring the deactivation of CuZn/SiO_2_ until the steady-state was achieved. Considering the selectivity trends, we observe that our most-active catalyst (CuZn/Al_2_O_3_) is the least selective for methanol (selectivity towards methanol is ca. 58%, at 250 °C, 40 bar) with significant production of dimethylether (DME) at the highest reaction temperatures (250 °C, 280 °C) and to a lesser extent of CH_4_, as shown in Fig. 5a and Supplementary Figure 7. This is due to the presence of the acidic sites of γ-Al_2_O_3_, which are able to further catalyze the dehydration of the produced methanol to DME^38,39^. Its monometallic counterpart, the Cu/Al_2_O_3_ catalyst, shows higher DME and CH_4_ selectivity, at the expense of methanol, and the minor (1%) production of longer hydrocarbons, such as C_2_H_6_. On the other hand, the silica- and ZnO/Al_2_O_3_-supported catalysts are highly selective toward methanol. Owing to the low amount of Cu and Zn with respect to the amount of the support material in our samples (<5 wt.% total concentration), the contribution from the support is more significant for our catalysts as compared with the industrial bulk-like samples (see the methods section for details).

## Discussion

Our XAS data acquired at relevant industrial pressures allow the pressure effect on the local structure of the CuZn/SiO_2_ catalyst to be determined. By comparing the results obtained during the activation under hydrogen (1 bar) and reaction steps at 20 and 40 bar, both from the XANES and EXAFS data analyses (Supplementary Figures 9 and 11 and Supplementary Table 8), it can be seen that the Cu structural parameters for the activation step (1 bar, 325 °C) are similar to those obtained under the methanol synthesis mixture at the highest temperature studied (40 bar, 320 °C, step that corresponds to the ageing treatment performed). Therefore, we conclude that no significant pressure or chemical potential effect can be detected on the Cu local structure for the CuZn/SiO_2_ catalyst. Combining these XAS results with the XRD data of the catalysts after reaction (these techniques are sensitive to short and long-range order, respectively), the majority of copper remains stable (XAS) but a small fraction of the NPs sinter (XRD and TEM). It is worth mentioning here that the after reaction TEM and XRD data were recorded after more than 150 h under operation, including steps at 280 °C, which could favor the aggregation of Cu. In addition, the XRD data also confirmed the formation of the CuZn phase on SiO_2_ after reaction (see Supplementary Figure 6 and Supplementary Discussion).

The temporal evolution of the catalytic performance displayed in Fig. 5b, where the CuZn/SiO_2_ gradually deactivates until it reaches the steady state after ca. 200 h under operation, can be linked to the gradual reduction of ZnO under operando conditions, as inferred from XAS and corroborated by NAP-XPS. As stated earlier, from the spectroscopic results under methanol synthesis conditions we observe the partial reduction of ZnO, leading to the coexistence of ZnO with reduced Zn species. Furthermore, from the NAP-XPS results, we can conclude that ZnO and reduced Zn species are mostly located at the surface of our CuZn NPs. By XAS, we observe a similar evolution of Zn after 20 h under 10% H_2_/He at 245 °C and 1 bar pressure (Fig. 3c). Thus, we can conclude that the partial reduction of ZnO and formation of either metallic Zn or Zn–Cu bonds are not dependent on the pressure or the specific composition of the reducing reaction (H_2_ versus CO_2_+CO+H_2_) mixture. Zn reduction is, however, significantly enhanced at higher temperatures (>250 °C). The temperature effect is also demonstrated in the aging experiment performed under 10% H_2_/He at 1 bar pressure and 245 °C, 320 °C, 450 °C, and 600 °C (Fig. 3d and Supplementary Figure 17), where the contribution of the Zn–M feature is low at 320 °C, but is increasingly more pronounced at 450 °C and 600 °C, demonstrating and emphasizing the temperature effect on the reducibility of the Zn species. The unambiguous attribution of the latter species to metallic Zn or CuZn alloy formation is not possible owing to their relatively low contribution to our XAS data, together with the similar scattering cross-sections of Cu and Zn, making them indistinguishable by XAS. However, the EXAFS Zn–M bond length is similar to the Cu–Cu bond length in metallic Cu, thus, the incorporation of Zn into a Cu-rich alloy is a viable hypothesis. Note that the formation of CuZn alloy (brass) in CuZn catalysts was previously reported under CO/H_2_ atmosphere at 600 °C^17^, and other references also suggested that some alloying of Cu and Zn can take place at temperatures as low as 250 °C^26^. Our results confirm that this process also takes place at lower temperatures, namely 245 °C, although then the rate of ZnO reduction is slower.

Regarding the catalytic activity, higher intrinsic methanol formation rates have been reported for Cu/Al_2_O_3_ than for Cu/SiO_2_^39^, which is in agreement with our data and has been attributed to the Lewis acid nature of the Al_2_O_3_ support. Nevertheless, the presence of the stronger acid sites of γ-Al_2_O_3_ leads to the dehydration of methanol to DME. From our data, we observe that the addition of Zn promotes the methanol formation for both, the CuZn/Al_2_O_3_ and the CuZn/SiO_2_ catalysts and its selectivity in the case of CuZn/Al_2_O_3_ versus Cu/Al_2_O_3_. This is remarkable and highlights the importance of the intimate CuZn interaction for the CO_2_+CO hydrogenation reaction, since in our catalysts zinc is not present as a bulk support, as done in previous studies, but as a highly diluted phase forming the CuZn NPs. For CuZn/SiO_2_, the addition of Zn causes a methanol production rate increase ca. three times with respect to its monometallic counterpart, and a similar increase is also obtained for CuZn/Al_2_O_3_. It is interesting to note here that the Zn K-edge XAS data revealed for the CuZn/Al_2_O_3_ catalyst the migration of Zn atoms to the alumina support and the lack of significant Zn reduction. Nevertheless, our data clearly show that Zn is still able to promote the methanol formation rate and improve its selectivity from 35% for Cu/Al_2_O_3_ to 58% for CuZn/Al_2_O_3_. By comparing the methanol yields of our NPs with those of the bulk-like industrial Cu/ZnO/Al_2_O_3_ catalyst reference measured under identical conditions (Supplementary Figure 8), a clear size-effect is observed, with a lower methanol production for the 2–3 nm NPs employed here, as previously reported elsewhere^4^. This result highlights that operating with the highest possible surface area materials (smallest NPs <5 nm) is not always beneficial for the catalytic process. In addition, the lower Cu and Zn concentrations used in our study (<5 wt.% total metal loading) could also lead to lower methanol productions than those obtained with the industrial catalyst^40^.

We finally conclude that morphologically, structurally, and chemically well-defined catalysts composed of ca. 3 nm Cu and CuZn NPs supported on ZnO/Al_2_O_3_, γ-Al_2_O_3_, and SiO_2_ were synthesized in order to investigate CuZn and NP/support interactions. By using operando XAS data acquired at high pressure (up to 40 bar), we were able to follow the evolution of the NPs’ structure and composition under reaction conditions. For both, CuZn/SiO_2_ and CuZn/Al_2_O_3_, the majority of the zinc species are present in the form of a disordered oxide, whose structure depends strongly on the support. In particular, the incorporation of Zn in the Al_2_O_3_ support was observed for CuZn/Al_2_O_3_, forming a spinel-like structure that hinders the reduction of the cationic Zn species under reaction conditions. On the other hand, for CuZn/SiO_2_, we observed the gradual reduction of the oxidic zinc species under reaction conditions, and the formation of Zn–Cu or Zn–Zn bonds. Although this reduction is accelerated at higher temperatures, it was already observed at temperatures commonly used for methanol production (i.e., 250 °C). The slow reduction of ZnO species in this sample observed by XAS is paralleled by a decrease in methanol yield as a function of the reaction time. After an initial deactivation, the steady-state methanol production of CuZn/SiO_2_ is still similar to that of Cu/ZnO/Al_2_O_3_, but CuZn/SiO_2_ is far more active than Cu/SiO_2_. This indicates the beneficial effect of the CuZn interaction even if the ZnO species get partially reduced or if they are present highly diluted as part of the NPs. In addition, NAP-XPS data revealed Zn surface segregation for CuZn/SiO_2_, and the formation of a distorted ZnO_x_ overlayer, virtually independent from the chosen reactant mixture. Our data indicate that a strong interaction of Cu and ZnO is necessary for a highly selective and active catalyst, which is affected by the choice of the support and the nature of the CuZn interface.

## Methods

### Synthesis of powder catalysts

The size-controlled NPs were synthesized by an inverse micelle encapsulation method. Monodisperse micellar NP solutions were prepared by dissolving [poly(styrene)-block-poly-(2-vinylpyridine), PS-P2VP, Polymer Source Inc.] polymers in toluene and stirred for 2 days. In parallel, the metal salts CuCl_2_·2H_2_O (Sigma Aldrich), ZnCl_2_ (Alfa Aesar), or Zn(OAc)_2_ (Sigma Aldrich) were dispersed in tetrahydrofuran and stirred for 2 days. Nominal Cu:Zn=70:30 atomic ratios were used. Subsequently, the solutions containing the metal salts were incorporated into the micellar solutions and stirred for 2 additional days. The PS-P2VP molecular weights and the polymer-head (P2VP)-to-metal salt ratios are reported in Supplementary Table 1.

The NP height was determined via AFM (Bruker MultiMode 8 microscope) on dip-coated SiO_2_/Si(111) wafers after polymer removal by oxygen plasma (0.3 mbar) (Fig. 1a and Supplementary Figure 1).

The Cu and Cu_0.7_Zn_0.3_ NPs were supported on nanocrystalline powders via incipient wetness impregnation of the NP solutions. The supports employed are commercial SiO_2_ (STREM chemicals), γ-Al_2_O_3_ (Inframat Advanced Materials), and ZnO/Al_2_O_3_ (containing 10% mol Al_2_O_3_) synthesized from the precipitation of Zn and Al following a variation of the method reported elsewhere^41^. For this, 12.34 g Zn(CH_3_CO_2_)_2_ and 2.52 g Al(NO_3_)_2_·9H_2_O were dissolved in 60 ml deionized water. Then 1 M Na_2_CO_3_ was added dropwise until a pH value of 9 was reached. After 1 h, the solution was washed, filtered, and dried to obtain a white powder. In the final step, the powder was calcined at 600 °C. XRD analysis showed only reflections corresponding to zincite (ZnO).

Temperatures for calcination and cleanliness (removal of polymeric carbon) after the calcination of the NPs supported on the oxide powders were determined by thermogravimetric analysis (TGA) (see Supplementary Figure 2). The catalysts were calcined for 6 h in a rotating tubular oven under a flow of 20% O_2_ in Ar at the temperatures obtained from the TGA analysis.

The metal content of the synthesized powders (after calcination) was determined by inductively coupled plasma-mass spectrometry (ICP-MS). To prepare the samples for ICP, a precise amount of the calcined powder catalyst was dissolved in 10 ml of a 1:1:3 mixture of H_2_SO_4_, HNO_3_, and HCl. This solution was digested in a microwave (Anton Paar GmbH, Multiwave GO) at 180 °C for 30 min. Then, the solution was further diluted with water. The results of the ICP measurements are given in Supplementary Table 2.

### TEM imaging

TEM and STEM images of the samples before and after reaction were acquired using the microscopes at the Fritz Haber Institute (Thermo Fisher Talos F200X, JEOL ARM200F, operated at 200 kV) in Berlin, at the Ernst Ruska-Centrum (FEI Titan 80-200, operated at 200 kV) in Jülich, and at the Ruhr-University (JEOL JEM-2800) in Bochum. The samples after reaction were imaged by transferring them under inert atmosphere to a glove box and loading them onto Au grids with a holey carbon film. The samples were transferred to the Talos TEM using a vacuum transfer holder. This procedure ensured no exposure to air/O_2_ after the reaction. For STEM analysis with a Cs-probe corrected FEI Titan 80-200 microscope, a probe semi-angle of 25 mrad and an inner collection semi-angle of the detector of 88 mrad were used to achieve high-angle-annular dark-field conditions. Compositional maps were obtained with EDX using four large-solid-angle symmetrical Si drift detectors. Additional STEM EDX maps are shown in Supplementary Figures 3–5.

### XRD characterization

The XRD patterns were recorded using a Bruker-AXS D8 Advance diffractometer equipped with a Cu K_α_ source and a position-sensitive energy-dispersive LynxEye XE-T detector. XRD patterns were recorded in continuous scanning mode in a 2θ range of 20–90 °, applying an increment 0.02 ° and a variable divergence slit configuration ensuring constant sample illumination.

Rietveld refinement was performed using the software package TOPAS^®^ (Bruker-AXS) to analyze the diffraction patterns taking into account instrumental broadening, zero error, and sample displacement. Owing to the structural complexity of the Al_2_O_3_, no Rietveld refinement was performed on the CuZn/Al_2_O_3_ diffraction pattern. Furthermore, the diffraction signals of the SiO_2_ support were considered as convolution of individual peaks, which made a Rietveld quantification impossible. The results of the XRD experiments are shown in Supplementary Figure 6 and Supplementary Tables 3–6.

### Catalytic testing

The catalytic activity was measured in a high-pressure fixed-bed flow reactor. About 50 mg of the catalyst were diluted with ~300 mg SiC (ratio 6:1) and then placed in a glass-lined steel tube. Before testing, all catalysts were reduced by flowing 10% H_2_ in He for 2 h at 245 °C. The activity was measured at pressures of 20, 40, and 60 bar and temperatures of 220 °C, 250 °C, and 280 °C. The reaction gas mixture consisted of a 10% CO, 4% CO_2_, 72% H_2_, and 14% He, which was used as an internal standard. The total flow was 17 ml/min. The reaction products were measured online by gas chromatography (GC) with an Agilent Technologies 7890B gas chromatograph equipped with a flame ionization detector and two thermal conductivity detectors. All reported values are the average of at least three consecutive injections. A carbonyl trap was used to ensure that the catalyst remains Ni-free. Our carbonyl trap consisted of a stainless steel tube filled with SiC, which was heated up to 300 °C. The trap was placed on the CO line directly before the mixing with the other gases took place. The absence of Ni after reaction was confirmed by TEM-EDX.

The values for the methanol production are normalized using the Cu content (grams of Cu) in the powder catalyst determined by ICP-MS. Supplementary Figure 7 shows additional results obtained during the catalytic measurements. The activity for each catalyst was measured in multiple consecutive reaction steps. After the reduction, the catalyst was cooled down to 220 °C and the reactant mixture was introduced, then the reactor was pressurized to 20 bar. The reactivity was then measured at 220 °C, 250 °C, and 280 °C, each temperature step lasting for 8 h. After this the catalyst was cooled down to 220 °C, before going to the next pressure. This procedure was repeated for the 40 bar and 60 bar data points, for a complete cycle of all reaction steps.

During the initial reaction steps, the catalytic activity was not stable, and a long activation period (50–140 h) was needed until the steady-state operation was achieved. The steady-state data for all catalysts are included in the main text (Fig. 5a) and in Supplementary Figure 7. The activity of the Cu/SiO_2_ and Cu/ZnO/Al_2_O_3_ catalysts was found to become stable relatively fast, since already during the step at 40 bar no major changes were observed. For the catalysts supported on γ-Al_2_O_3_, the stabilization took longer time, and the catalyst only showed stable methanol production values during the 60 bar reaction step. During the activation of the Cu/SiO_2_, Cu/ZnO/Al_2_O_3_, Cu/Al_2_O_3_, and CuZn/Al_2_O_3_ catalysts, the production and selectivity toward methanol increases and they reach a steady state after the first cycle of all reaction conditions. The data displayed in Fig. 5 shows the results of a second cycle for all reaction conditions once the steady state was reached. However, the activation period is longer for the CuZn/SiO_2_ catalyst than for the others, as it does not become stable during the first run through all reaction temperatures and pressures. For this reason, we stayed at one reaction condition (280 °C and 40 bar) until a steady state was reached, which in this case took 150 h (see Fig. 5b). The same was done for the CuZn/Al_2_O_3_ catalyst for comparison (see Fig. 5b). Using the knowledge gained from the operando XAS experiments, we can conclude that this slow change, which is accelerated at higher temperatures is related to the formation of a metallic Zn phase. Another unique feature of the activation of the CuZn/SiO_2_ catalyst is that the methanol production first increases with time, as was the case for all the other catalysts, but then only for this catalyst the activity decreases after ~50 h. The subsequent reduction of the catalytic activity of the CuZn/SiO_2_ sample can then be associated with the reduction of Zn, which was only found to take place for this catalyst. The values given in Fig. 5a and Supplementary Figure 7 are collected after the activation period, during the steady-state operation, when no changes in activity over time were observed.

The empty reactor was also tested before each experiment to ensure the absence of any background contribution or residuals from previous measurements. In this test, the empty reactor was heated up and pressurized while flowing the reaction gas mixture. To further ensure the reliability of the measurements carried out here on low-metal loading NPs, a commercial copper-based methanol synthesis catalyst (Alfa Aesar, 45776) with a sieve fraction 100–200 μm, was tested under the same conditions in our reactor, showing the high selectivity and yield expected for this high-copper loading sample (Supplementary Figure 8).

### Operando XAS

The operando XAS measurements were performed at beamline 2–2 at SSRL (operando high-pressure experiments) and Quick X-ray Absorption and Scattering (QAS) beamline at NSLS-II synchrotron (ambient pressure/high temperature control experiments). Beryllium tube reactors (for measurements at pressures up to 40 bar) and quartz capillary reactors (for measurements at pressures up to 20 bar) were used to emulate the packed bed reactors used for the catalytic tests. The reactors were loaded with the sample, which was fixed in position with quartz wool plugs. The reactor was connected to a gas manifold, consisting of multiple mass flow controllers for precise gas dosing. A backpressure regulator adjusted the pressure in the reactor. The beryllium reactor was heated with a tubular oven with a window for the beam. This configuration only allows for measurements in transmission. The quartz reactors were heated with heating coils and enables, dependent on the configuration, measurements in fluorescence. The samples were diluted with boron nitride to optimize the absorption of the sample for transmission measurements. Control experiments at the QAS beamline were carried out in fluorescence mode using a PIPS detector. A 30% detuned Si(220) double crystal monochromator was used for energy selection at SSRL, whereas a Si(111) monochromator was used at NSLS-II. The ionization chambers used as X-ray detectors for transmission measurements were filled with N_2_. Cu and Zn reference foils were also measured together with the spectra of the samples for energy calibration.

During the operando studies at SSRL, all samples were initially measured under He atmosphere and at room temperature to record their initial state. Subsequently, the 10%H_2_/He mixture was dosed and the temperature was raised until the reduction temperature was achieved, which was kept for 2 h. The reduction temperature was 245 °C for the Cu/ZnO/Al_2_O_3_ and the CuZn/Al_2_O_3_ catalysts and 325 °C for the CuZn/SiO_2_ catalyst. A higher activation temperature was used for the sample deposited on SiO_2_ in order to reduce the CuO_x_ species, which were found to be more stable on this support. In a separate follow-up experiment (see below), we used 245 °C activation temperature also for the CuZn/SiO_2_ catalyst and obtained similar results as for the sample activated at 325 °C, thus the difference in activation temperatures is not crucial here.

Next, the temperature was lowered to 220 °C and the reaction mixture (10% CO, 4%CO_2_, 72% H_2_, 14% He) was introduced and the reactor was pressurized to the required working pressures, i.e., 20 and 40 bar. Heating rates of 5 °C/min were used for all temperature changes. The reaction series consisted of: (i) 20 bar at 220 °C, (ii) 20 bar at 280 °C, (iii) 40 bar at 280 °C, and (iv) 40 bar at 320 °C. The last step at high temperature (320 °C) was performed in order to mimic an aging treatment. A mass spectrometer recorded the gaseous effluent of the reactor.

For the CuZn/SiO_2_ sample, we performed additional experiments where the sample was activated at 245 °C in a H_2_/He mixture at 1 bar for 2 h. The sample was then cooled down to room temperature, and XAS spectra were recorded at room temperature to obtain high-quality data that are not obstructed by high thermal disorder. Subsequently, the sample was exposed to reaction conditions (220 °C and 20 bar) for 2 h, then depressurized and cooled down to room temperature, where XAS spectra were also recorded.

Moreover, in a separate experiment we followed the reduction of the CuZn/SiO_2_ sample during a long-term treatment, where the sample was kept at 245 °C in a H_2_/He mixture (at atmospheric pressure) for 20 h. Afterwards, the sample cell was cooled down to room temperature, where XAS spectra were collected.

Finally, at the QAS beamline we also performed control experiments for the CuZn/SiO_2_ sample, where the sample was kept in a H_2_/He mixture at 1 bar, and the temperature was gradually increased from 245 °C to 320 °C, then to 450 °C and 600 °C, to enhance the reduction of the Zn species.

### In situ NAP-XPS

The NPs for the NAP-XPS study were synthesized using the same method described above, using a PS-P2VP (48,500:70,000) polymer to obtain the micelles. Deposition of the particles on the SiO_2_/Si(100) support was done by dip coating (1 cm/min). The polymer ligands were removed by an O_2_ plasma treatment (SPI Plasma Prep III Plasma Etcher, 20 min, 20 W, 350 mTorr). The procedure of dip-coating and plasma treatment was repeated three times to increase the density of particles on the support. The particle size and distribution on the SiO_2_/Si(100) was measured by AFM. The NP height values were obtained with the open-source software Gwyddion (Supplementary Figure 19).

The NAP-XPS measurements with synchrotron radiation were performed at the HIPPIE beamline of the MAX IV synchrotron in Lund (Sweden) and the IOS beamline at NSLS-II in Brookhaven (NY, USA). Additional lab-based NAP-XPS measurements were carried out at the FHI Berlin using Al K_α_ radiation and a hemispherical analyzer (Phoibos 150, SPECS GmbH). The synchrotron measurements were performed at X-ray energies of 1250 eV and 1580 eV to obtain a depth profile, as different kinetic energies result in different escape depths of the photoelectrons. 1250 eV corresponds to an IMFP of ~0.8 nm for Cu *2p* and 0.7 nm for Zn *2p* photoelectrons, probing the outermost layers of the NPs, and 1580 eV corresponds to ~1.2 nm for Cu *2p* and Zn *2p*, thus probing deeper regions^33^. The energy values were chosen to avoid overlap with peaks resulting from the Auger electrons. The LMM X-ray generated Auger regions for Cu and Zn were also recorded. All peaks were aligned to the elemental Si *2p*_3/2_ peak at a BE of 99.4 eV. The Si peak itself was fitted using a doublet, with an energy splitting of 0.6 eV. For the quantification of the elemental composition at given photon energy, the peaks of the Cu *2p*_3/2_ and Zn *2p*_3/2_ regions were fitted. The obtained values were corrected with the relative sensitivity factors of the different elements. For quantitative analysis of the peak area, the IMFP and ionization cross-section for the corresponding photon energy were taken into account.

The series of NAP-XPS measurements were as follows: the first step was oxidation in pure O_2_ (0.13 mbar at 400 °C) to remove any adventitious carbon on the surface of the sample. The C *1* *s* peak was monitored during the oxidation and the experiment only continued when no traces of carbon were detected. This was followed by a reduction in H_2_ (1.3 mbar, 350 °C) for the activation of the catalyst by reduction of the Cu and afterwards, the reaction gas mixture (1.3 mbar, 250 °C) was introduced. The measurements were carried out under different reaction mixtures, whose concentrations are shown in Supplementary Table 7. Between each of these steps, the sample was cooled down in the gas atmosphere to near room temperature, then the chamber was evacuated, and filled with the new gas before heating up. For each reaction mixture, a new measurement series was performed introducing an identically prepared but fresh sample.

An important aspect to be considered when synchrotron NAP-XPS investigations are conducted is the strong influence of the X-ray beam on the sample (Supplementary Figure 20). During the measurements in a gas atmosphere, besides the reversible surface segregation discussed in the main text, irreversible radiation-induced segregation trends were also observed. In fact, we detected the loss of the metal segregating to the surface (e.g., Cu in O_2_ atmosphere, and Zn in H_2_ atmosphere and under the reaction gas mixture) when the X-ray beam stayed on the same spot of the sample surface for several minutes. To avoid any radiation-induced effects on our results, the spot on the sample was changed to a new, fresh location after each set of scans and the Cu *2p* and Zn *2p* regions were measured in alternating order in consecutive rounds of measurements. The reproducibility and reliability of the results presented here was confirmed in a large number of independent measurements at the synchrotron, as well as analogous investigations with a lab-based NAP-XPS system.

## Supplementary information

Supplementary Information

## Data Availability

All data are available from the authors on reasonable request.
