# Supplementary material for: Operando high-pressure investigation of size-controlled CuZn catalysts for the methanol synthesis reaction
Source: Nat Commun. 2021 Mar 4;12:1435. doi: 10.1038/s41467-021-21604-7 (PMC7933282; doi:10.1038/s41467-021-21604-7)
Supplement: Supplementary file 1 — Supplementary Information [file 41467_2021_21604_MOESM1_ESM.pdf]

Supplementary Information for

Operando High-Pressure investigation of Size-Controlled CuZn Catalysts for the Methanol Synthesis Reaction

Núria J. Divins[1⊥†], David Kordus[1,2†], Janis Timoshenko[2], Ilya Sinev[1], Ioannis Zegkinoglou[1], Arno Bergmann[2], See Wee Chee[2], Simon Widrinna[1,2], Osman Karslıoğlu[2], Hemma Mistry[1], Mauricio Lopez Luna[2], Jian Qiang Zhong[2], Adam S. Hoffman[3], Alexey Boubnov[3], J. Anibal Boscoboinik[4], Marc Heggen[5], Rafal E. Dunin-Borkowski[5], Simon R. Bare[3], Beatriz Roldan Cuenya[2]*

Correspondence to: roldan@fhi-berlin.mpg.de

This PDF file includes:

Supplementary Discussion

Supplementary Figures 1 to 25

Supplementary Tables 1 to 9

Supplementary References

Supplementary Discussion

XRD

X-ray diffraction (XRD) patterns of the $Cu/ZnO/Al_2O_3$, $CuZn/SiO_2$ and $CuZn/Al_2O_3$ catalysts were recorded in their as-prepared calcined state as well as after reaction (Supplementary Figure 6). A main CuO phase was observed on all as-prepared samples, together with $Cu_3Zn(OH)_6Cl_4$ for $CuZn/SiO_2$ and $CuZn/Al_2O_3$ and $Zn_5(OH)_8Cl_2 \cdot H_2O$ for $Cu/ZnO/Al_2O_3$. The residual Cl from the metal salt precursor was not observed after the reaction. Rietveld refinement of the as-prepared $CuZn/SiO_2$ and $Cu/ZnO/Al_2O_3$ diffraction patterns revealed crystalline domain sizes mostly larger than 5 nm, indicating that some sintering took place during the calcination. XRD is, however, not sensitive to highly disordered small NPs, and therefore, we mainly observe the agglomerated NPs. In the case of the $CuZn/Al_2O_3$, only support diffraction peaks could be seen after reaction, which indicates the formation of small, non-crystalline NPs. In $CuZn/SiO_2$, the lattice parameter of the main fcc phase agrees well with a slightly expanded Cu lattice suggesting minor (<5 %) Zn incorporation. Additionally, some Zn segregated after reaction, forming a ZnO phase. A secondary Cu-rich phase with expanded lattice formed also in $Cu/ZnO/Al_2O_3$.

Operando XAS data

The Cu K-edge XAS data obtained are shown in Supplementary Figures 9 (Cu K-edge XANES), 10 and 11 (Cu K-edge EXAFS), as well as in Figure 2 in the main text. At the Cu K-edge, *operando* XAS spectra collected at different conditions are almost indistinguishable and only minor changes due to temperature-dependent thermal disorder can be observed.

The Zn K-edge XAS data obtained are shown in Supplementary Figures 14 (Zn K-edge XANES), 15 and 16 (Zn K-edge EXAFS), as well as in Figure 2 in the main text.

a) EXAFS data for the Cu K-edge

EXAFS spectra of the samples in their initial state (after calcination) resemble those for bulk CuO. In particular, the first peak in the FT-EXAFS spectra at ~1.5 Å (uncorrected for phase shift) can be associated with Cu—O bonds. During the activation in hydrogen and under reaction conditions, a clear Cu—M (here M is Cu or Zn) shell corresponding to metallic Cu develops at ca. 2.24 Å (uncorrected for phase shift) for all catalysts. In agreement with the XANES data, contributions of Cu—O bonds are not detectable for any of the catalysts under reaction conditions, and all the spectra of the three catalysts resemble strongly that of metallic Cu. Slight changes in the main FT-EXAFS peak intensity observed under reaction conditions (Supplementary Figure 11) correlate well with the corresponding changes in temperature and can be attributed to thermal disorder effects. It is also worth noting that the Cu K-edge EXAFS of all catalysts contain pronounced contributions from distant coordination shells (peaks in FT-EXAFS at large *R* values), which indicate the presence of relatively ordered Cu phase that could be attributed to our micelle encapsulation synthesis and the long calcination pre-treatment performed in 20% O_2/inert at 400°C to remove the polymer before the activation in H_2 and the reaction.

To characterize the local structure of the investigated catalysts quantitatively, EXAFS data fitting for the first coordination shell was performed. Conventional least-square fitting to theoretical standards, as implemented in the FEFFIT code[1] was applied. Theoretical phases and amplitudes were obtained in self-consistent ab-initio calculations with the FEFF8.5 code[2] for bulk

Cu and CuO materials. The complex exchange-correlation Hedin-Lundqvist potential and default values of muffin-tin radii as provided within the FEFF8.5 code were employed.

We started with single shell fitting of the Cu foil and CuO data to obtain the values of the amplitude reduction factors S_0^2. The obtained values were used later for the fitting of the experimental EXAFS data for nanocatalysts. Fitting of spectra for reference materials were carried out in the same ranges in *k*- and *R*-spaces as the ones later used for the nanocatalysts, to partially compensate for systematic errors due to the limited signal length in *k*-space.

For CuO and for as-prepared catalysts (initial state) we fit only the first peak in the Fourier-transformed EXAFS spectra, which in bulk CuO corresponds to 4 nearest oxygens[3]. More distant peaks contain overlapping contributions of both Cu—M and Cu—O pairs, as well as contributions of multiple-scattering effects, thus they are challenging to interpret in conventional analysis. Fitting of EXAFS spectra $\chi(k)k^2$ thus is carried out in *R*-space in the range from $R_{\min}$ = 1.0 Å up to $R_{\max}$ = 2.0 Å. Fourier transform was carried out in the *k* range from 3.0 Å^{-1} up to 9.5 Å^{-1}. Fitting parameters were coordination numbers *N* and interatomic distances *R* and disorder factors σ^2 for Cu—O bonds, and corrections to photoelectron reference energies ΔE_0.

For all of the catalysts under reaction conditions, a very good fit can be obtained by including a Cu—M contribution only. Addition of Cu—O contribution did not improve the fit significantly, and the Cu—O coordination number (CN) was in all cases equal to 0 within uncertainties. The fitting of the EXAFS spectra $\chi(k)k^2$ was carried out in *R*-space in the range from $R_{\min}$ = 1.0 Å up to $R_{\max}$ = 3.0 Å. Fourier transform was carried out in the k range from 3.0 Å^{-1} up to 9.5 Å^{-1}.

For the quantitative EXAFS data fitting of the samples during the reduction in hydrogen, we used the same fitting model as for the sample under reaction conditions but included also a Cu—O contribution.

The results of Cu K-edge EXAFS data fitting are summarized in Supplementary Figures 12 and 13 and Supplementary Table 8. In addition to EXAFS data fitting, for the reduced samples we performed also the analysis of the coordination numbers using an advanced interpretation of the XANES data based on an artificial neural network (NN) approach[4]. The results of the NN-XANES analysis are also shown in Supplementary Table 8.

For catalysts in their initial state the obtained structure parameters values are in very good agreement with those for bulk CuO, which also agrees with the conclusions from the visual examination of the EXAFS data. Only a small reduction in Cu—O CNs was detected, and this effect is close to the uncertainty of our analysis. During the reduction treatment in hydrogen, the CN for the Cu—O bond gets close to 0, while the Cu—M CN increases, indicating the reduction of all samples. In addition, the σ^2 factors that can be associated with static and thermal disorder, increase due to the temperature increase.

For the three catalysts under reaction conditions, the obtained Cu—M coordination numbers are ca. 8 – 10, in a good agreement with the NN-XANES results, and are slightly reduced with respect to the bulk Cu value, 12. The observed CNs values can be linked to a particle-size effect, and are in agreement with the expected particle size of ca. 3 nm. These results, and the lack of changes in the CN values under reaction conditions, suggest that while some sintering was observed in the XRD and TEM data (Supplementary Figure 3-6), the majority of Cu must be found within well-dispersed NPs that remain stable during the reaction.

The lower Cu-M CNs for the CuZn/Al_2O_3 sample could be assigned to a smaller average NP size, in agreement with our XRD data. Note, nevertheless, that the CN ca. 8, observed for the CuZn/Al_2O_3 sample, seems to be rather small for 3 nm particle size, and may suggest the presence of some heterogeneity in the sample (coexistence of particles of different sizes) or flattening of the nanoparticle shape due to strong particle-support interactions[5]. During the reaction, all the changes in the CNs are within the uncertainty of the analysis, suggesting that NPs were stable and their sizes did not change significantly under the reaction conditions tested. The pressure effect on the environment of Cu seems also to be negligible.

As obtained from the EXAFS data analysis, the interatomic Cu—M distances for all three samples are also similar, and slightly smaller than that for bulk copper. The apparent shortening of the interatomic distances in the *operando* data can be explained by an increase of anharmonicity in the atomic thermal motion at higher temperatures that results in skewed bond-length distributions not accounted for in our EXAFS analysis[6]. Indeed, when the samples after treatment are cooled down to room temperature, the Cu—M interatomic distance, yielded by the EXAFS data fit, agrees well with the Cu—Cu distance in metallic Cu (see Supplementary Table 8). Overall, when the results for the three catalysts are compared, in all cases the values obtained for the structural parameters are close, suggesting a weak dependency of the local structure around Cu due to the presence of Zn as part of the NPs or as a support.

b) EXAFS data for the Zn K-edge

The Zn K-edge EXAFS spectrum (see Figure 2b in the main text) of the as-prepared CuZn/SiO_2 catalyst only shows a prominent Zn-O feature at 1.55 Å (uncorrected for phase shift), revealing a high degree of structural disorder. During the activation and under reaction conditions,

an additional peak appears at ca 2.2 Å (Supplementary Figure 15) that can be linked to the formation of metallic Zn-Zn or Zn-Cu bonds, in agreement with the indications of minor sample reduction from the XANES analysis. The CuZn/Al_2O_3 catalyst also shows a strong Zn—O contribution in the first coordination shell. At the same time, no significant changes in the Zn K-edge EXAFS spectra can be observed under reaction conditions, except for the broadening of the features in the Fourier-transformed spectra due to an increase in the thermal disorder. In addition, the contribution of distant coordination shells is higher in this sample, which may be linked to the additional feature in the Zn K-edge XANES spectra (see arrow in Figure 2a), which indicates that Zn is forming an ordered phase with the alumina in the support. This strong interaction with the support may hinder the reduction of the Zn species under reaction conditions.

To obtain quantitative information, we performed Zn K-edge EXAFS data fitting. Similarly, as for the Cu K-edge, conventional least-square fitting to theoretical standards, implemented in FEFFIT code, was applied. Theoretical phases and amplitudes were obtained in self-consistent *ab-initio* calculations with FEFF8.5 code for bulk ZnO and for bulk Cu, where one Cu atom is replaced by Zn. The complex exchange-correlation Hedin-Lundqvist potential and default values of muffin-tin radii as provided within the FEFF8.5 code were employed. We started with the single shell fitting of the ZnO data to obtain the value of the amplitude reduction factor S_0^2. The obtained value was later on used for the fitting of the experimental EXAFS data of the nanocatalysts. Fitting of the spectrum for ZnO was carried out in the same range in *k*- and *R*-spaces as later used for the nanocatalysts, to partially compensate for systematic errors due to the limited signal length in *k*-space.

For ZnO we fit only the first peak in the Fourier-transformed EXAFS spectra, which in bulk ZnO corresponds to 4 nearest oxygens. Fitting of the EXAFS spectra $\chi(k)k^2$ is thus carried out in

R-space in the range from R_{min} = 1.0 Å up to R_{max} = 3.0 Å. Fourier transform was carried out in the k range from 3.0 $Å^{-1}$ up to 9.5 $Å^{-1}$. The fitting parameters were the coordination numbers N and the interatomic distances R, disorder factors σ^2 for Zn—O bonds, and a correction to the photoelectron reference energies ΔE_0.

Next, we employ a similar fitting procedure to analyze the Zn K-edge data of all nanocatalysts. In this case it was found that an inclusion of Zn—M (where M is Zn or Cu) contribution improves the fit quality. The obtained coordination numbers for this path in all cases are small. To reduce the uncertainties, we constrain the Debye-Waller factors for this path by the correlated Debye model: all spectra for the nanocatalysts are fitted simultaneously and the Debye-Waller factors for the Zn—M paths are calculated as $\sigma_s^2 + \sigma_{Debye}(T, \Theta)$, where σ_s^2 and Θ are fitting parameters (common for all samples), and T is the corresponding sample temperature. Also, a common value for Zn—O and Zn-metal distance was used for all spectra.

Zn K-edge EXAFS spectra obtained in follow-up experiments for a $CuZn/SiO_2$ sample reduced in H_2 at 245°C were fitted separately using an analogous fitting model. Only the data collected at room temperature (in the initial state, after the activation treatment and after sample exposure to reaction conditions) were included.

For the $CuZn/SiO_2$ sample in the initial state the local structure resembles that of bulk ZnO, since the Zn—O CN is close to 4. Upon the initial reduction treatment and under reaction conditions, the Zn—O CN slightly decreased, while Zn—M CN systematically increased, suggesting that the ZnO component of this catalyst gets gradually reduced and some fraction of metallic Zn was formed. The presence of Zn—M bond in the $CuZn/SiO_2$ sample was confirmed also by fitting the EXAFS data from follow-up experiments where the sample was cooled down to

room temperature after the reduction in hydrogen at 245°C and after exposure to the reaction conditions. The obtained Zn—M distance is similar to the Cu—Cu distance in metallic bulk copper, suggesting the formation of Cu-rich alloy.

In the as-prepared CuZn/Al_2O_3 catalyst, Zn is also 4-fold coordinated. The changes in the sample structure with increasing temperature are not pronounced for this sample. The reduced Zn—O CNs observed for CuZn/Al_2O_3 at high temperatures are likely an artifact due to the correlation between CNs and disorder factors, and the very short spectral *k*-range available for the analysis. Finally, by comparing the results obtained for CuZn/Al_2O_3 and CuZn/SiO_2 at different pressures, all structure parameters agree within error bars, and no significant pressure effect can be detected.

Additional NAP-XPS data, C *1s* and O *1s* spectra

C *1s* and O *1s* spectra were acquired during the NAP-XPS experiments. The peaks observed before the oxidation in the C *1s* region (Supplementary Figure 24) correspond to adventitious carbon. During the oxidation this peak decreased and the oxidation treatment was continued until it completely disappeared. During the methanol synthesis reaction, peaks corresponding to CO and CO_2 in the gas phase could be measured, but no additional features could be_unequivocally assigned, due to the low intensities.

The oxygen contribution from the SiO_2 support is the main contribution to the XPS signal in the O *1s* region. We could identify peaks caused by the gas phase reactants (O_2, CO_2, CO) in their respective mixture. A clear assignment of the oxidized Cu or Zn contribution could not be done because of the low metal NP loading (ca. 5% of the signal), which leads to the oxygen *1s* region

being dominated by the SiO_2 contribution. Additionally the ZnO and CuO_x features cannot easily separated since they share similar binding energies[7].

XPS and high-pressure reaction cell (HPC) experiments

To address the existing gap between the low pressure NAP-XPS measurements and the high pressure XAS experiments we performed additional investigations which show that for this material system, the findings extracted from the NAP-XPS data can be also extrapolated to high pressure reaction conditions. For this purpose, we used a laboratory-based UHV system equipped with NAP-XPS and a high pressure reaction cell (HPC) directly attached to it. The HPC is a cell placed inside a UHV chamber that can be closed and subsequently pressurized up to 20 bar with a chosen gas atmosphere, while being heated. Afterwards gas in the high pressure cell is pumped out and the sample is reintroduced back to the UHV environment without any air exposure. Then the sample is transferred through UHV directly to the XPS chamber where the XPS measurements are then conducted under UHV conditions. A picture of the experimental setup can be found in Supplementary Figure 25a.

Supplementary Figure 25b displays the findings of these experiments. The sample was first measured in UHV in its initial state before any treatment. Afterwards, adventitious carbon was removed from the sample in 1 mbar oxygen at 400°C, similarly as in the synchrotron NAP-XPS experiments, and the sample was re-measured afterwards. Then the sample is moved to the HPC where it is exposed to 1 bar of hydrogen and heated at 350°C for 2h. Afterwards, another XPS scan is performed in UHV. Then the sample is transferred to the HPC again, and exposed to 20 bar of H2+CO2+CO (84/4/12, same ratio used for the NAP-XPS synchrotron experiments for this mixture) and heated to 250°C for 2h. Next, the final XPS scan under UHV conditions is performed.

The depth profiling is performed in these laboratory-based experiments by comparing the *2p* and *3p* regions of Cu and Zn photoelectrons. Because of the higher binding energies of the *2p* electrons, their contribution is more surface sensitive. Clear surface segregation of Zn can be observed after the sample was exposed to the reaction gas mixture, closely resembling the behavior observed in the in situ NAP-XPS experiments. It should be noted that because an Al K-alpha source ($h\nu$=1487 eV) was used, the binding energies of the emitted electrons differ from those in the NAP-XPS synchrotron experiments and, therefore, the probing depths are slightly different from those in the synchrotron results. Nonetheless, the segregation trends remain the same. The results from this experiment show that the sample behaves in the same way under mbar pressure of the reactant mixture during the synchrotron NAP-XPS measurements and under 20 bar pressure during the HPC treatment. Thus, there is no pressure effect (in the range of pressures investigated) in the segregation trends.

Supplementary Figures

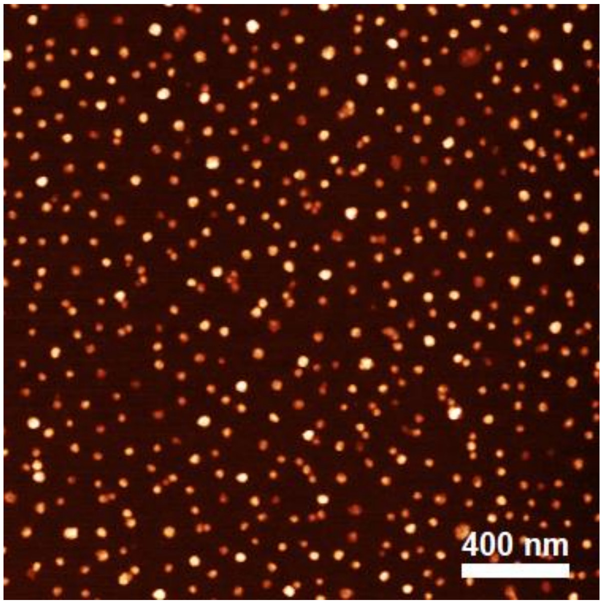

Supplementary Figure 1. Representative AFM image of the $Cu_{0.7}Zn_{0.3}$ NPs on SiO_2/Si(111)

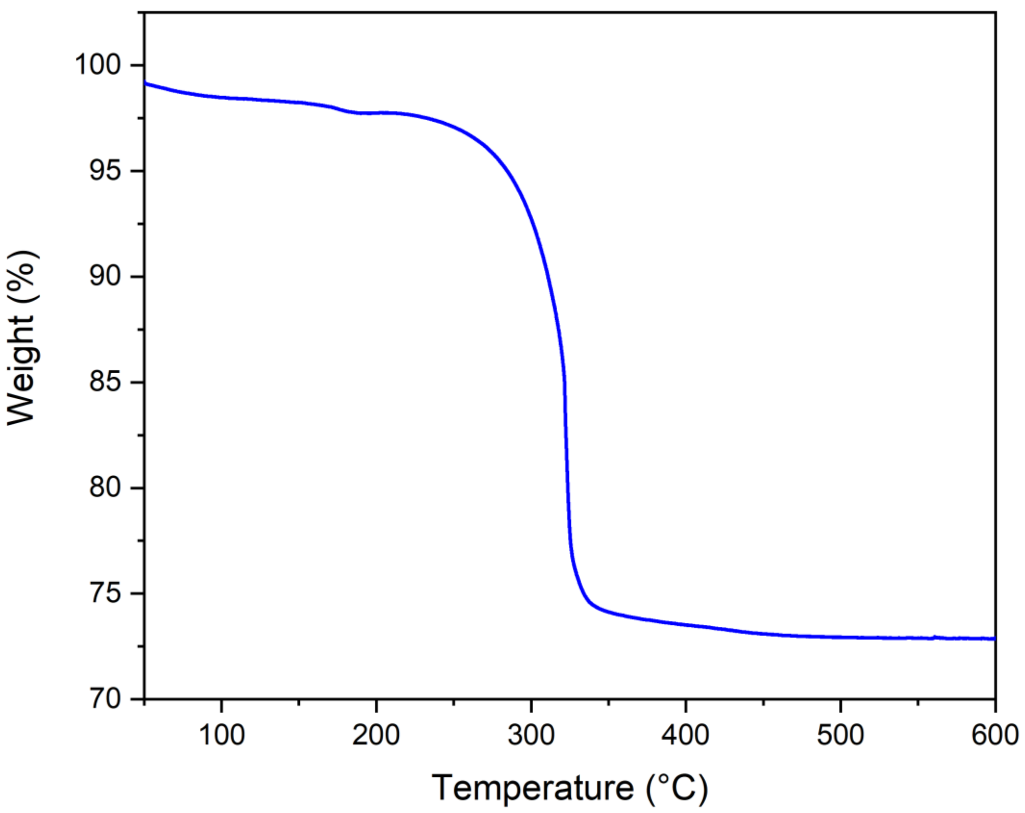

Supplementary Figure 2. Example of TGA measurement for a non-calcined catalyst, in this case the Cu NPs on SiO_2 (Cu/SiO_2 catalyst). The sample was heated from 50°C to 600°C with a ramp of 5°C/min in synthetic air. The weight loss corresponds to the removal of the polymer from the synthesis. When repeating the same experiment after the calcination, a flat curve is obtained. In the calcination applied to the samples used for the reactivity studies, the temperature was chosen accordingly to completely remove all of the polymer and was kept between 400℃ and 460℃.

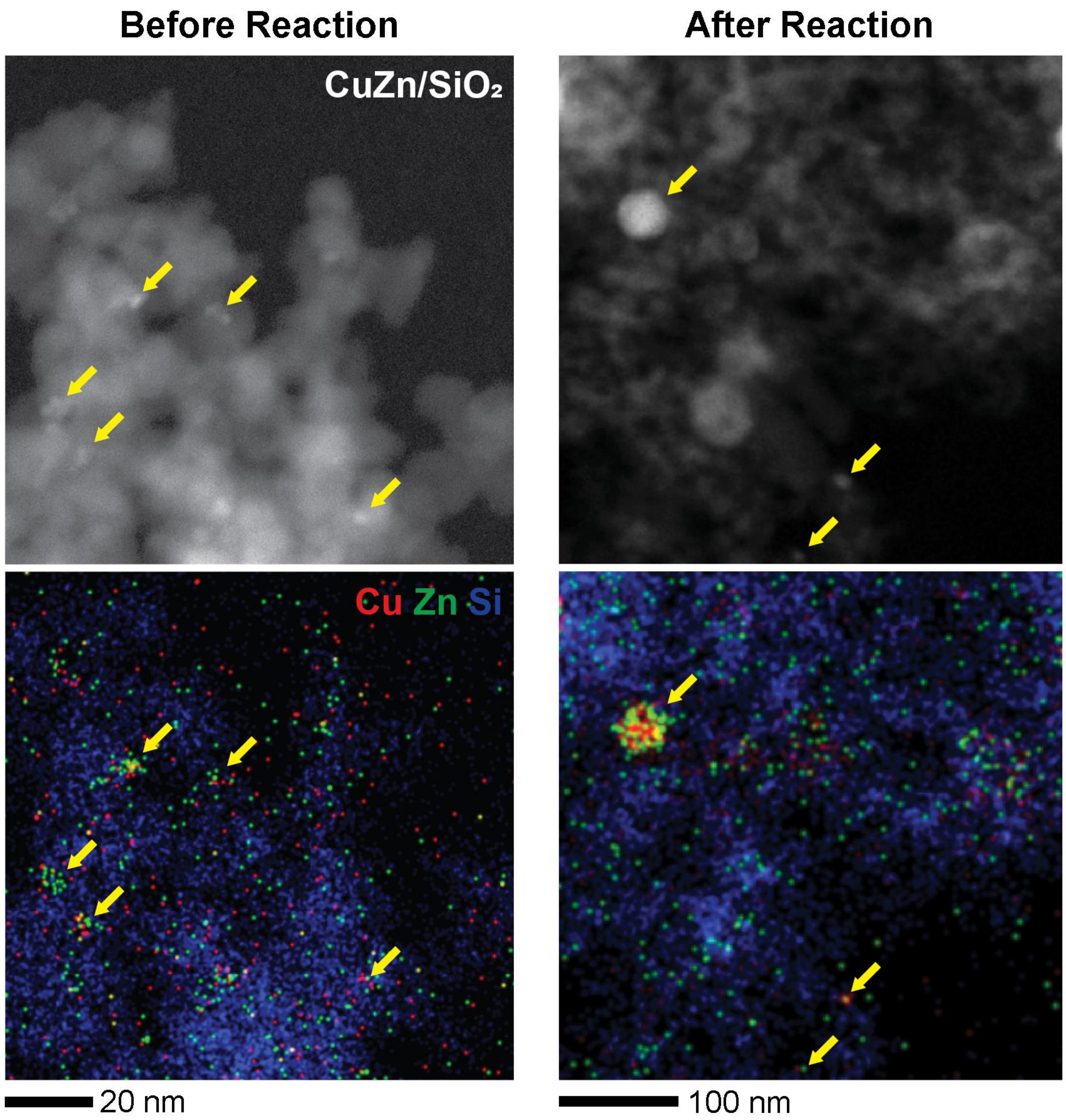

Supplementary Figure 3. STEM images and corresponding EDX maps of the CuZn/SiO_2 catalyst acquired before and after reaction. Selected NPs are highlighted with yellow arrows. In the EDX maps, Cu is shown in red, Zn in green and Si in blue.

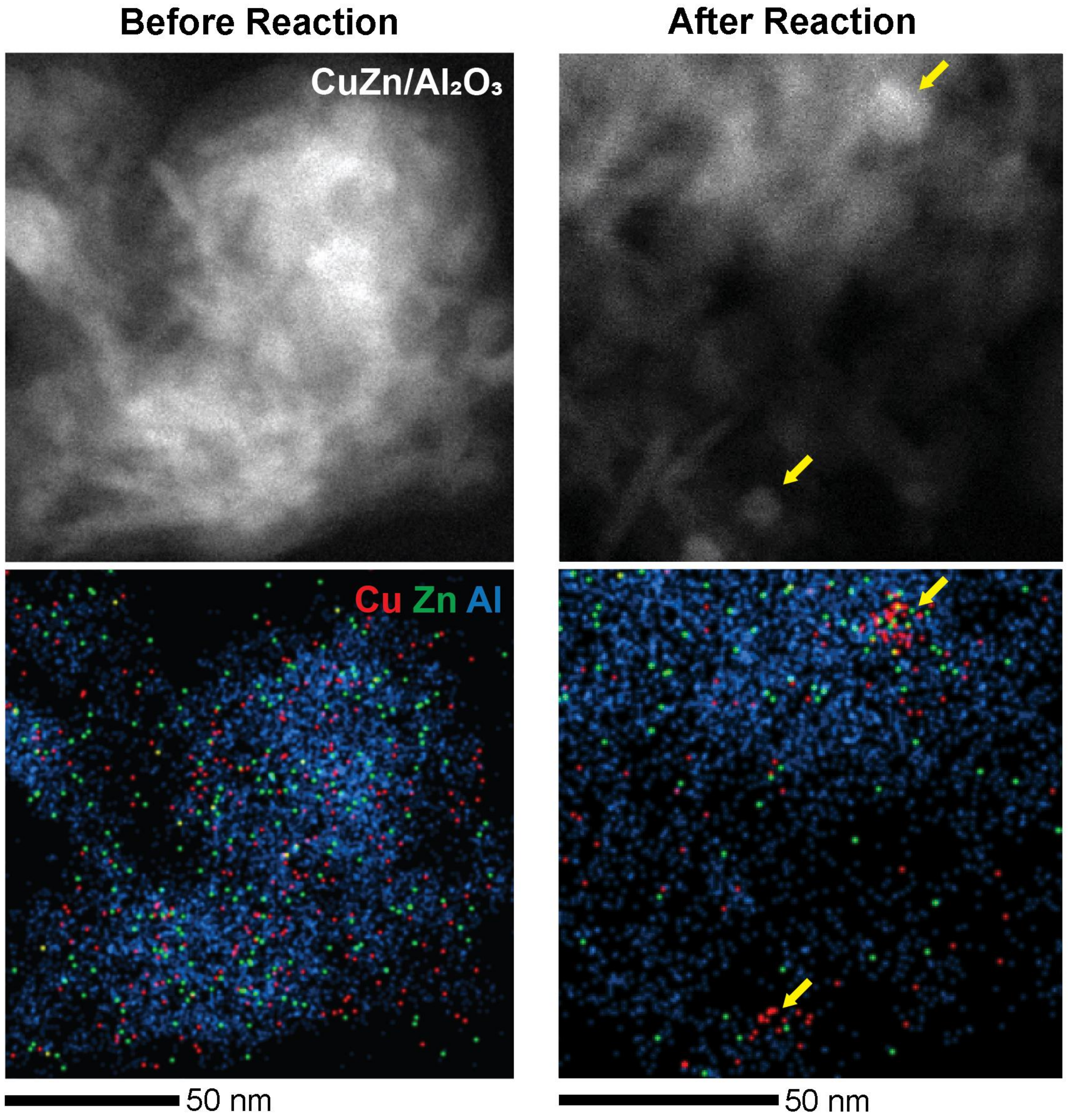

Supplementary Figure 4. STEM images and corresponding EDX maps of the $CuZn/Al_2O_3$ catalyst acquired before and after reaction. Selected NPs are highlighted with yellow arrows. In the EDX maps, Cu is shown in red, Zn in green and Al in blue.

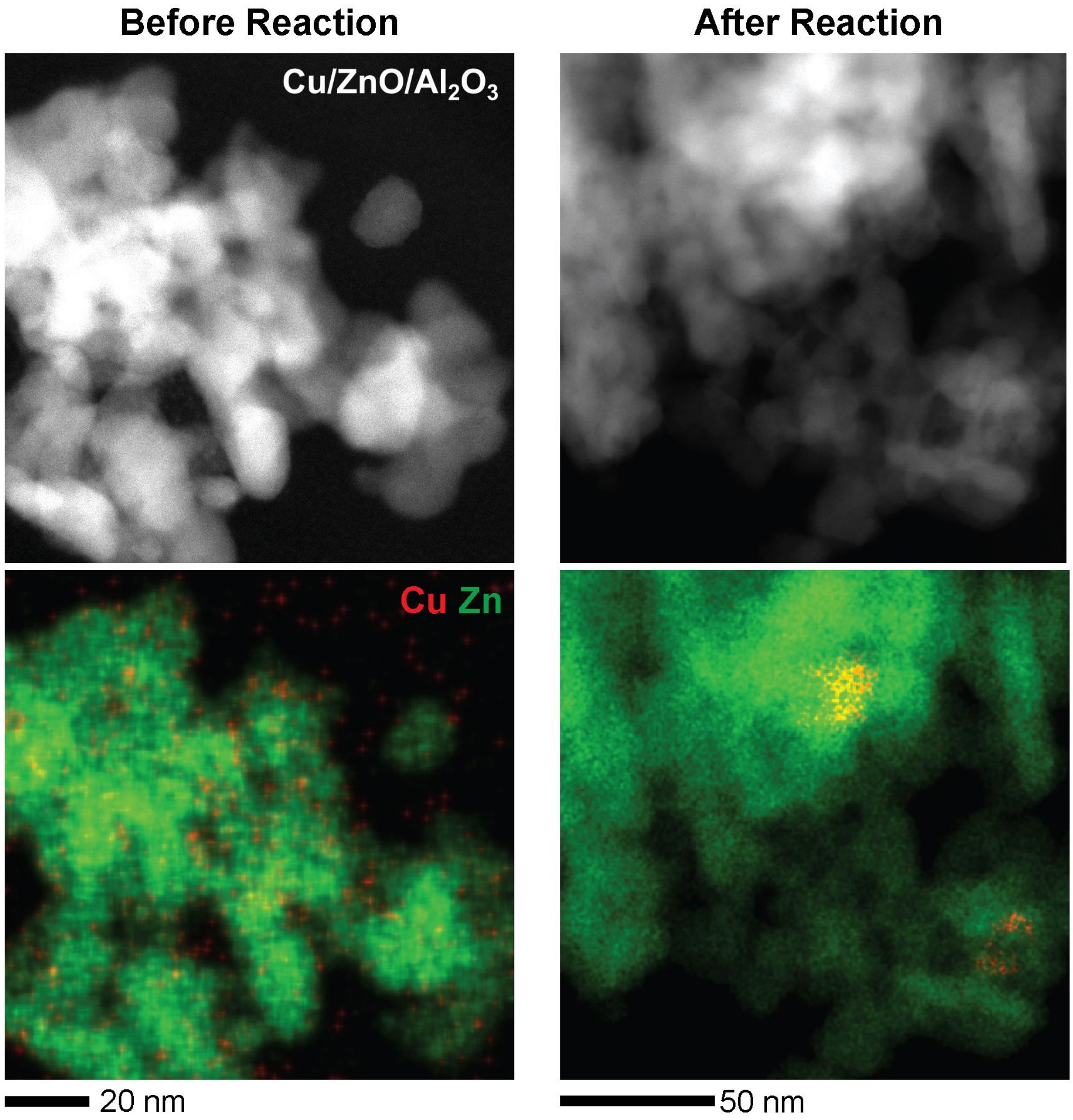

Supplementary Figure 5. STEM images and corresponding EDX maps of the $Cu/ZnO/Al_2O_3$ catalyst acquired before and after reaction. In the EDX maps, Cu is shown in red, Zn in green.

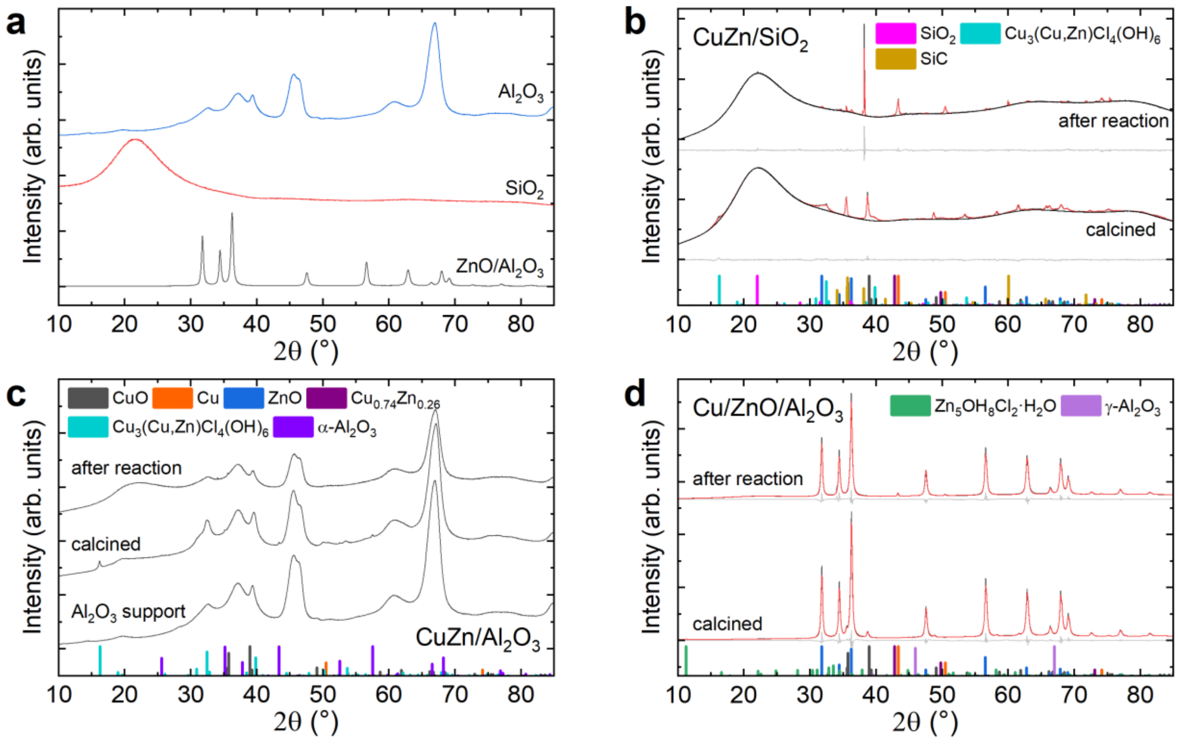

Supplementary Figure 6. X-ray diffractograms (black) of **a** the support materials, **b** CuZn/SiO_2, **c** CuZn/Al_2O_3, and **d** Cu/ZnO/Al_2O_3 in the calcined state and after reaction and the calculated profiles (red) achieved by Rietveld refinement. The bar plots show the peak positions of the reference pattern of the identified crystalline phases as extracted from the Inorganic Crystal Structure Database (ICSD).

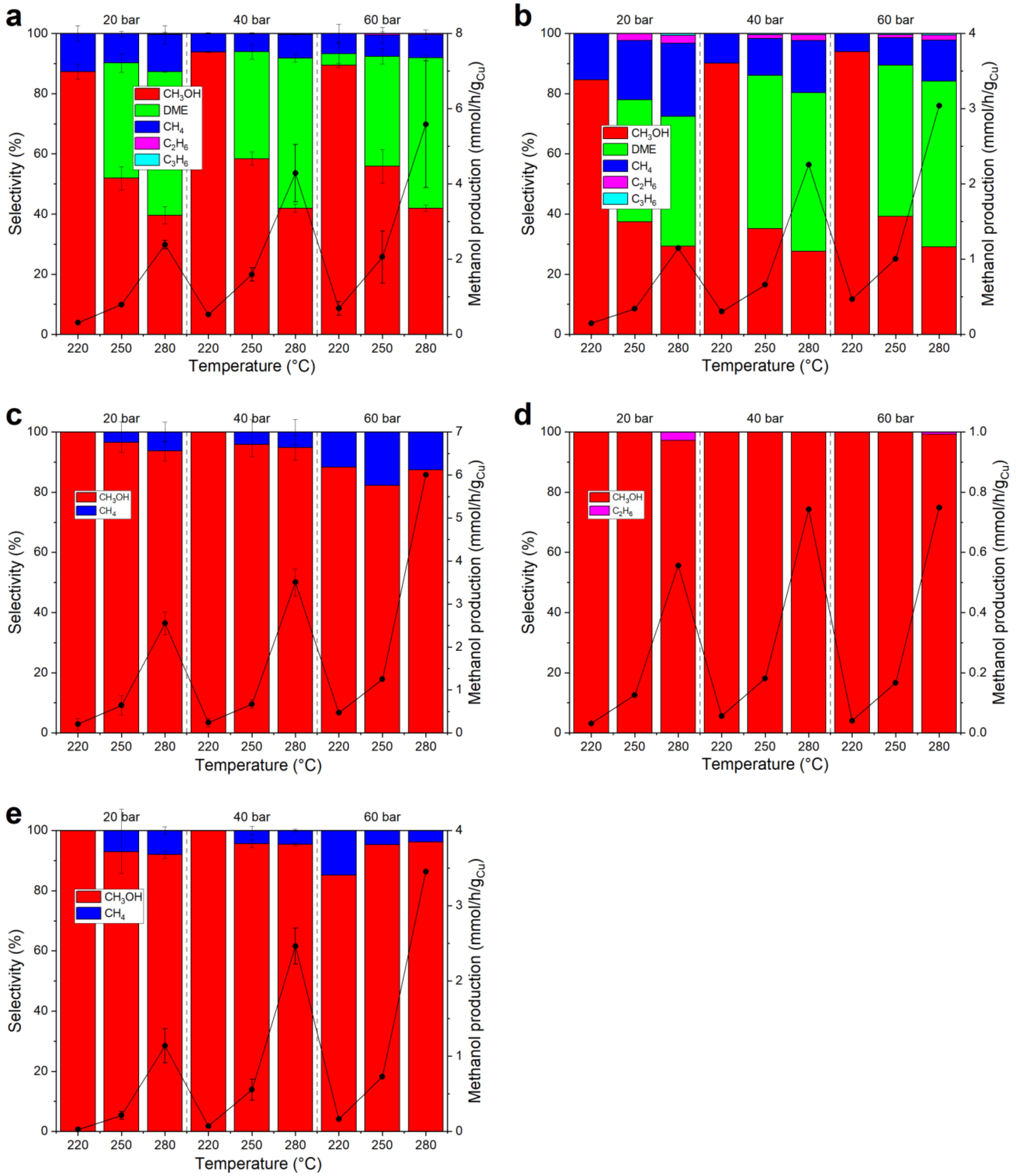

Supplementary Figure 7. Selectivity and methanol production obtained for the **a** CuZn/Al_2O_3, **b** Cu/Al_2O_3, **c** CuZn/SiO_2, **d** Cu/SiO_2 and **e** Cu/ZnO/Al_2O_3 catalyst measured at 20 bar, 40 bar and 60 bar as a function of increasing reaction temperature. Error bars correspond to the standard deviation.

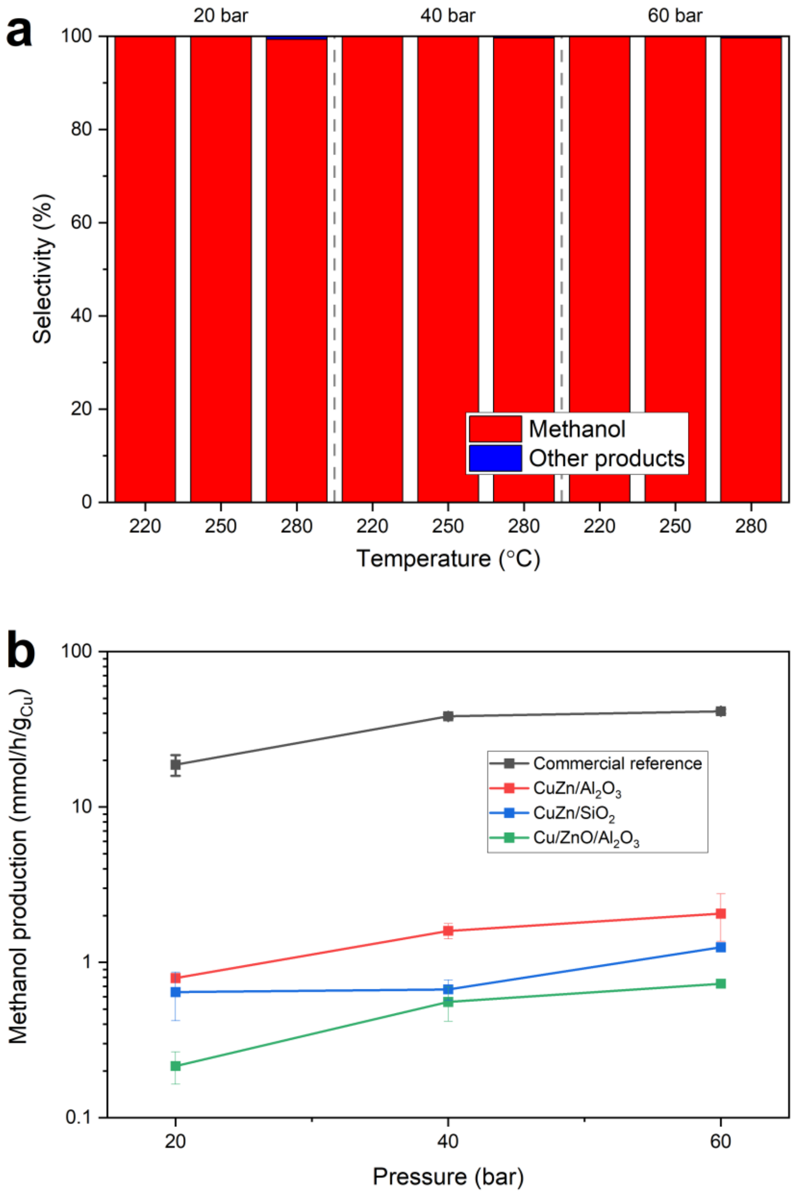

Supplementary Figure 8. Tests of the commercial catalyst done in our reactor setup. **a** Selectivity of the Catalyst shows a selectivity >99% for all reaction condition. With the same gas mixture and flow rate (17 ml/min) as for the other catalysts. Other products are mainly DME and CH_4. **b** Comparison of the activity obtained with the commercial reference with our catalysts (T=250°C). Error bars correspond to the standard deviation.

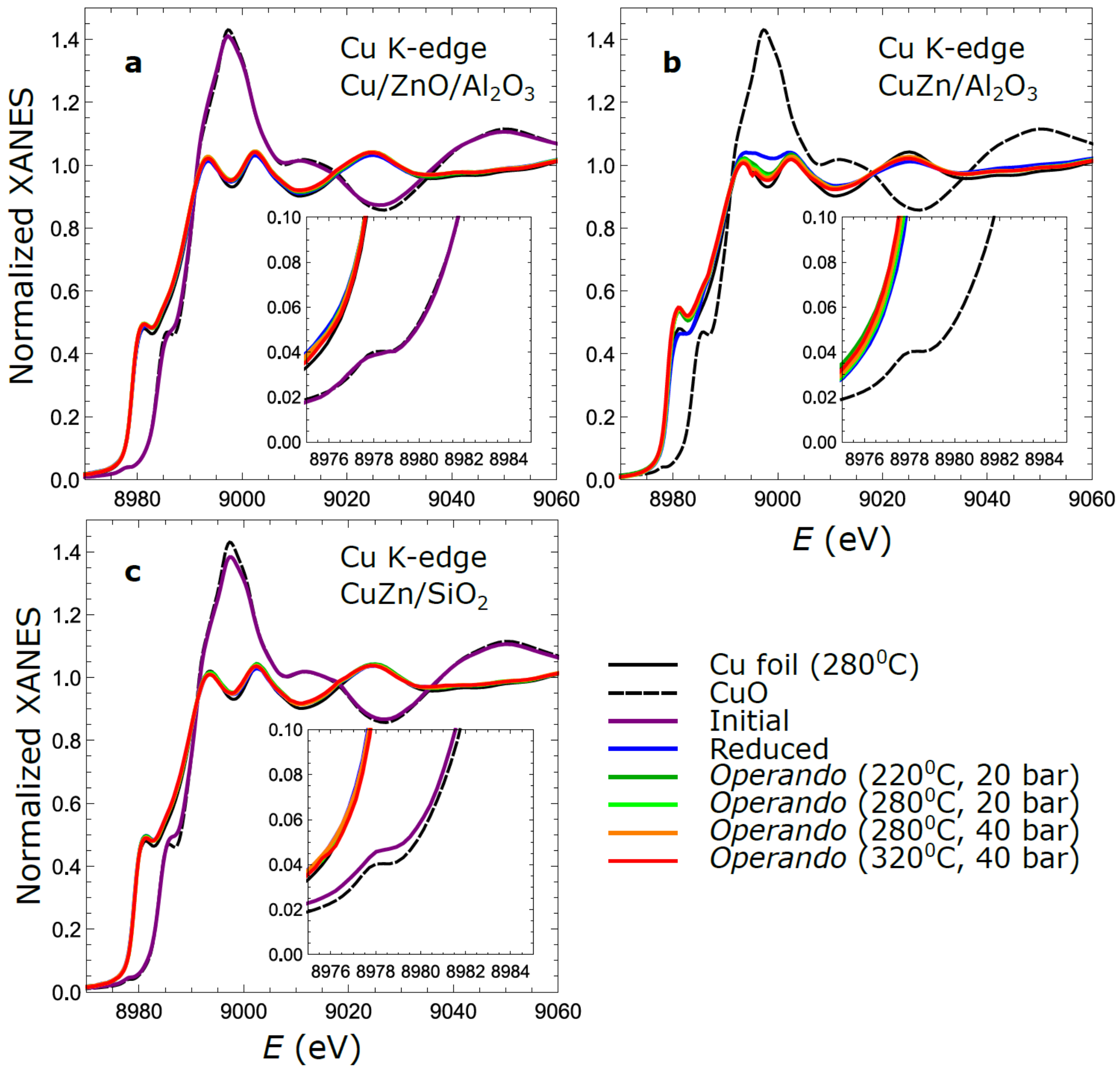

Supplementary Figure 9. *Operando* Cu K-edge XANES spectra of **a** Cu/ZnO/Al_2O_3, **b** CuZn/Al_2O_3 and **c** CuZn/SiO_2 catalysts under CO_2 hydrogenation conditions at the different temperatures and pressures tested, as indicated on the plots. The reference spectra of a metallic Cu foil and CuO are also shown.

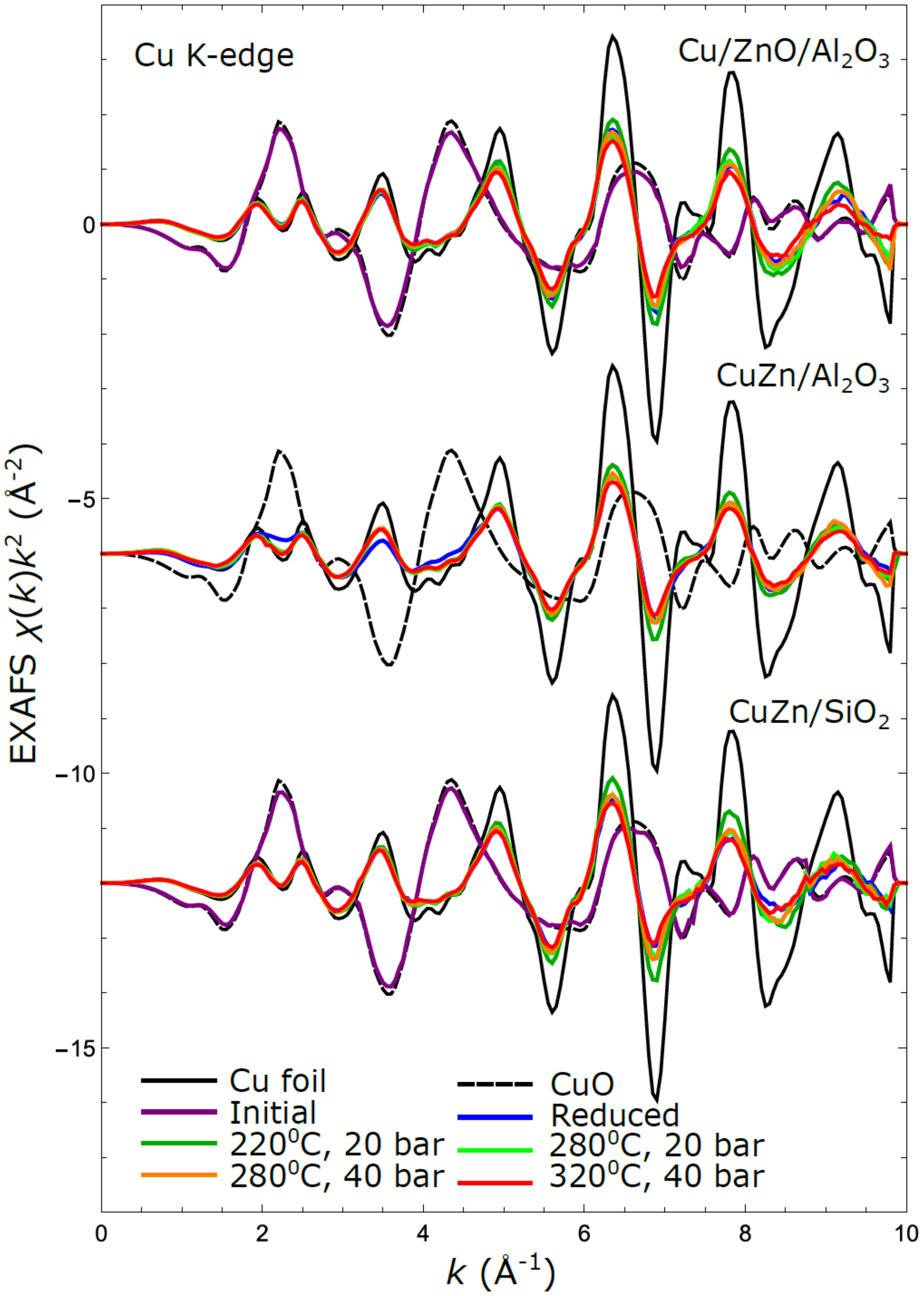

Supplementary Figure 10. *Operando* Cu K-edge EXAFS spectra ($\chi(k)k^2$) of Cu/ZnO/Al_2O_3, CuZn/Al_2O_3 and CuZn/SiO_2 catalysts under CO_2 hydrogenation conditions at the different temperatures and pressures tested, as indicated on the plots. The reference spectra of a metallic Cu foil and CuO are also shown. The spectra are shifted vertically for clarity.

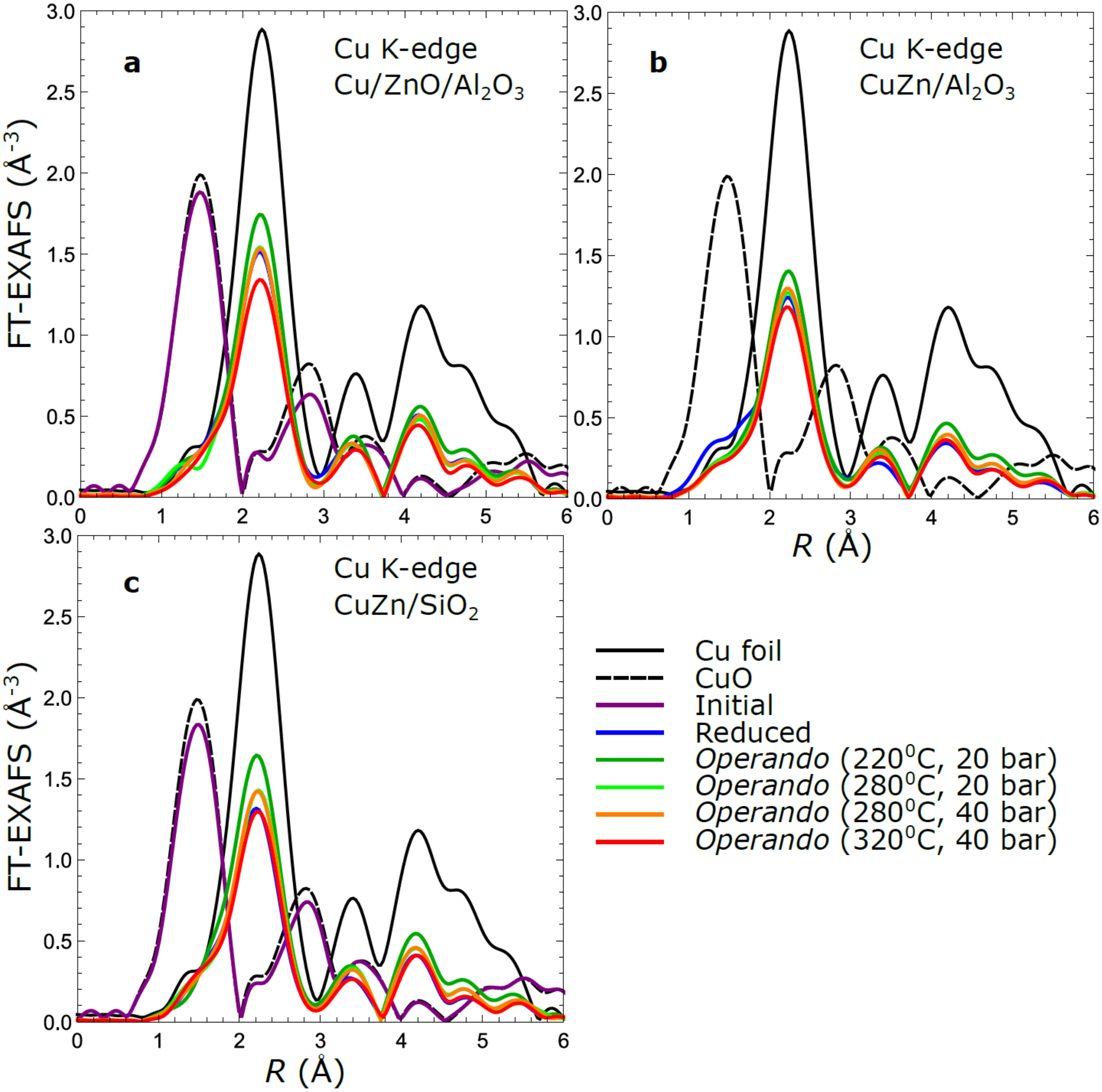

Supplementary Figure 11. Fourier-transformed *operando* Cu K-edge EXAFS spectra ($\chi(k)k^2$) of **a** $Cu/ZnO/Al_2O_3$, **b** $CuZn/Al_2O_3$ and **c** $CuZn/SiO_2$ catalysts under CO_2 hydrogenation conditions at different temperatures and pressures.

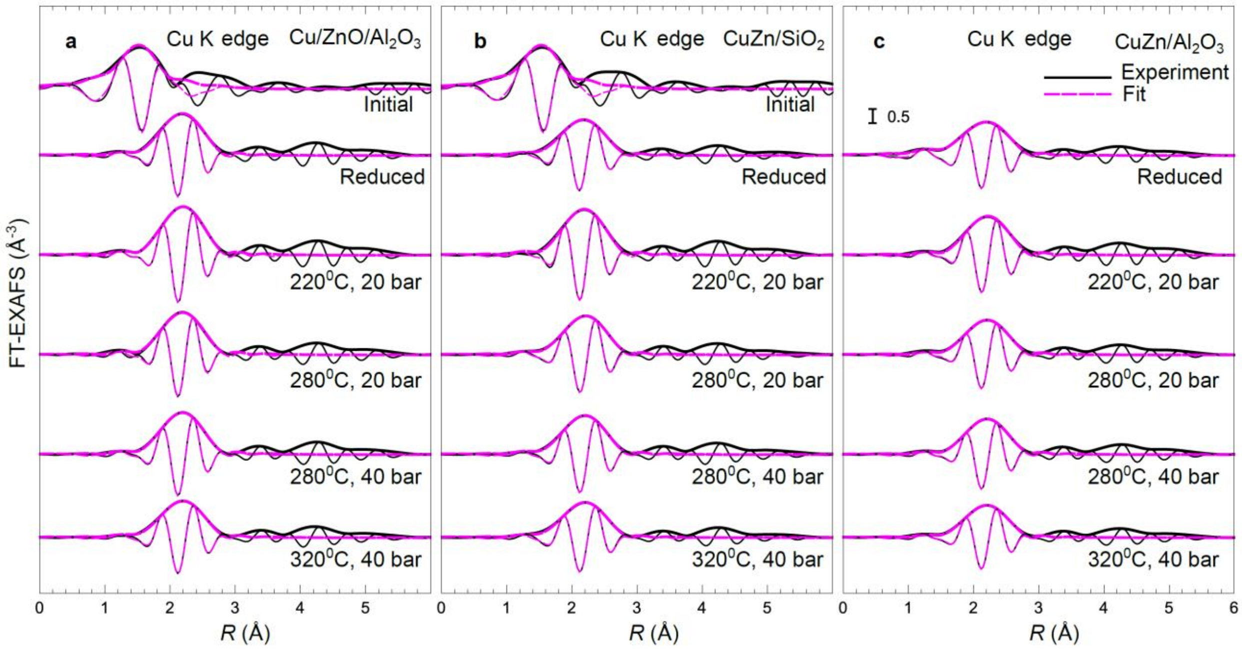

Supplementary Figure 12. Fits of Cu K-edge EXAFS spectra for **a** Cu/ZnO/Al_2O_3, **b** CuZn/SiO_2 and **c** CuZn/Al_2O_3 catalysts in their initial state at ambient conditions, during activation in hydrogen at T=245 °C for Cu/ZnO/Al_2O_3 and CuZn/Al_2O_3 catalysts, and T=325 °C for CuZn/SiO_2 catalyst, and in *operando* conditions at indicated temperatures and pressures. The fit range goes from 3.0 $Å^{-1}$ up to 9.5 $Å^{-1}$. Spectra are shifted vertically for clarity. Magnitudes (thick lines) and imaginary parts of FT-EXAFS spectra are shown.

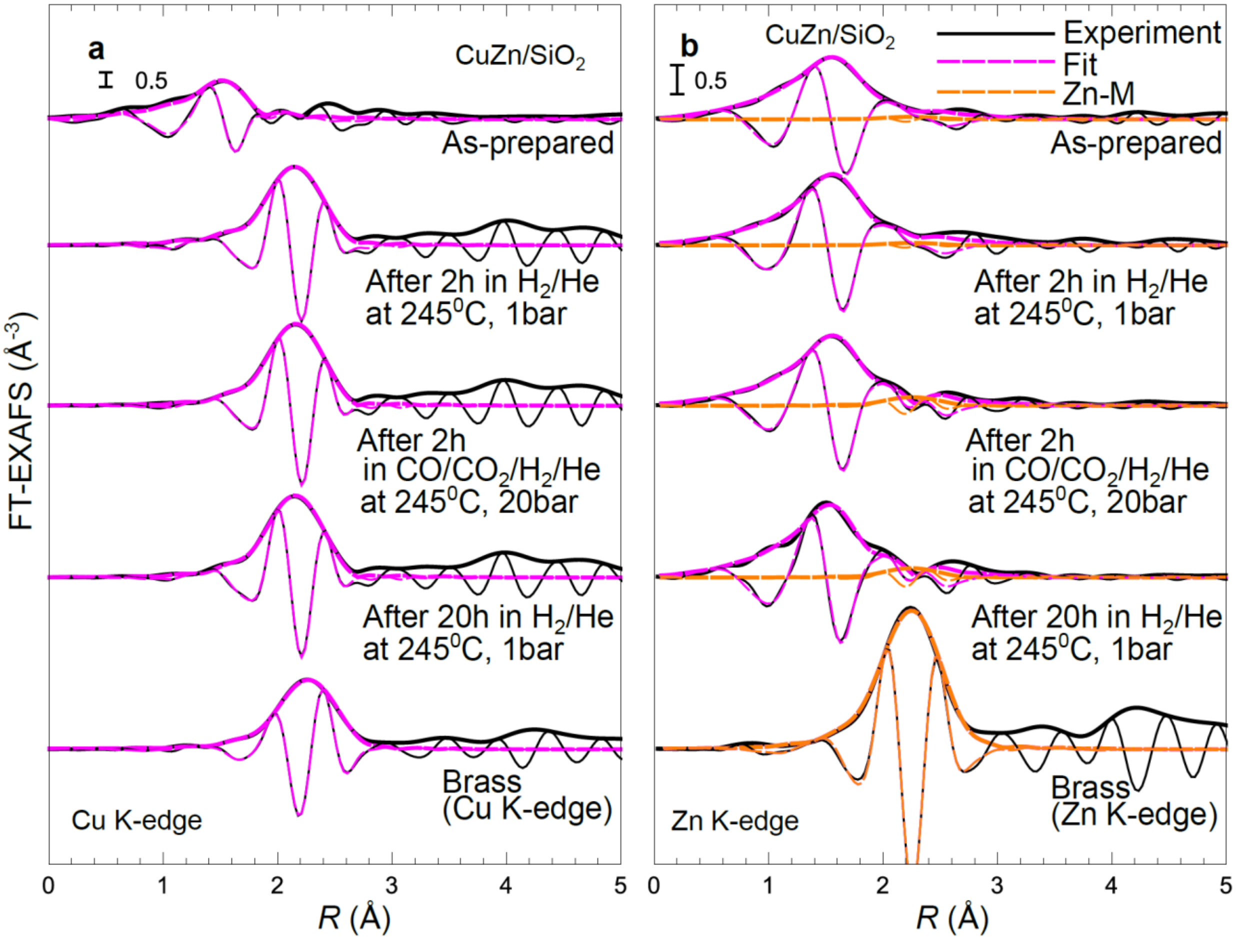

Supplementary Figure 13. Fits of **a** Cu K-edge and **b** Zn K-edge EXAFS spectra obtained in control experiments for CuZn/SiO_2 catalysts. All spectra are acquired at room temperature and 1 bar pressure after treatment under the conditions indicated on the plots. Spectra are shifted vertically for clarity. Magnitudes (thick lines) and imaginary parts of FT-EXAFS spectra are shown. The Zn—M contribution, obtained in the EXAFS data fitting, is shown separately. Fitting results for CuZn brass are shown for comparison.

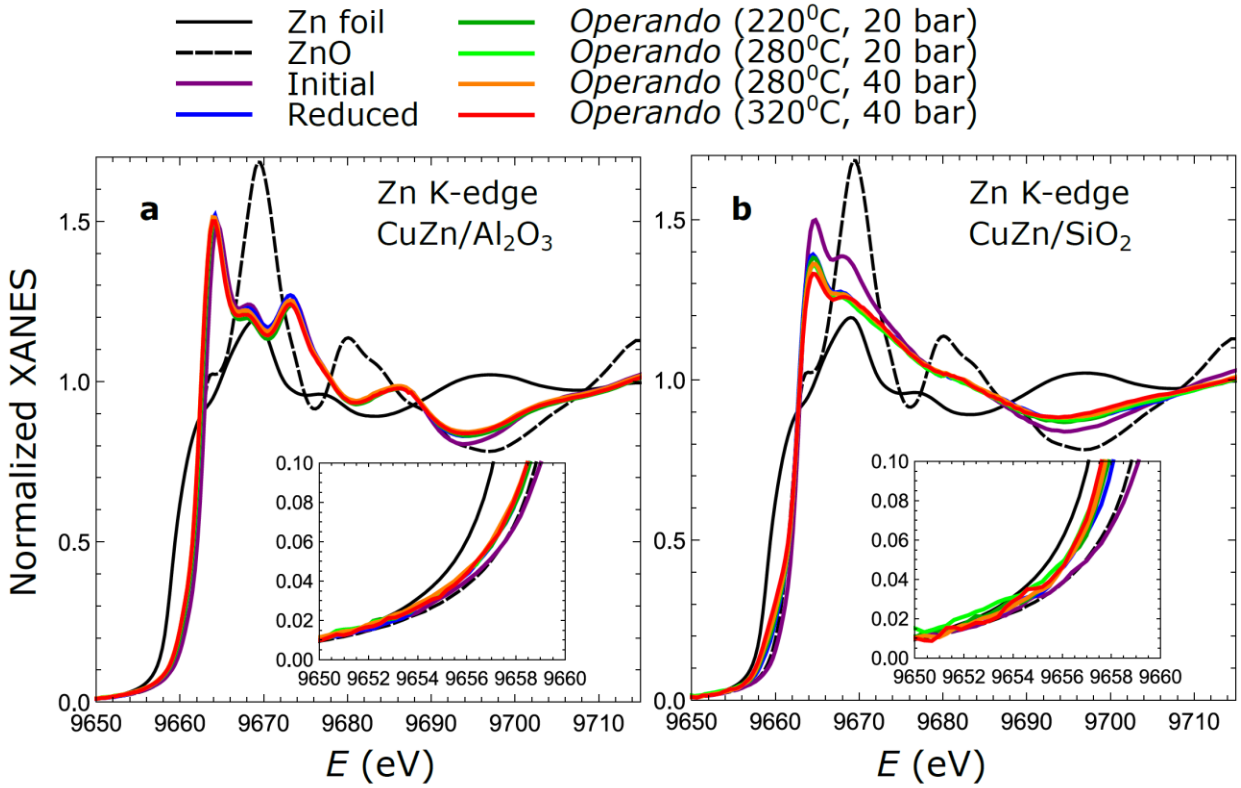

Supplementary Figure 14. *Operando* Zn K-edge XANES spectra of **a** CuZn/Al_2O_3 and **b** CuZn/SiO_2 catalysts under CO_2 hydrogenation conditions at the different temperatures and pressures tested, as indicated on the plots. The reference spectra of a metallic Zn foil and ZnO are also shown.

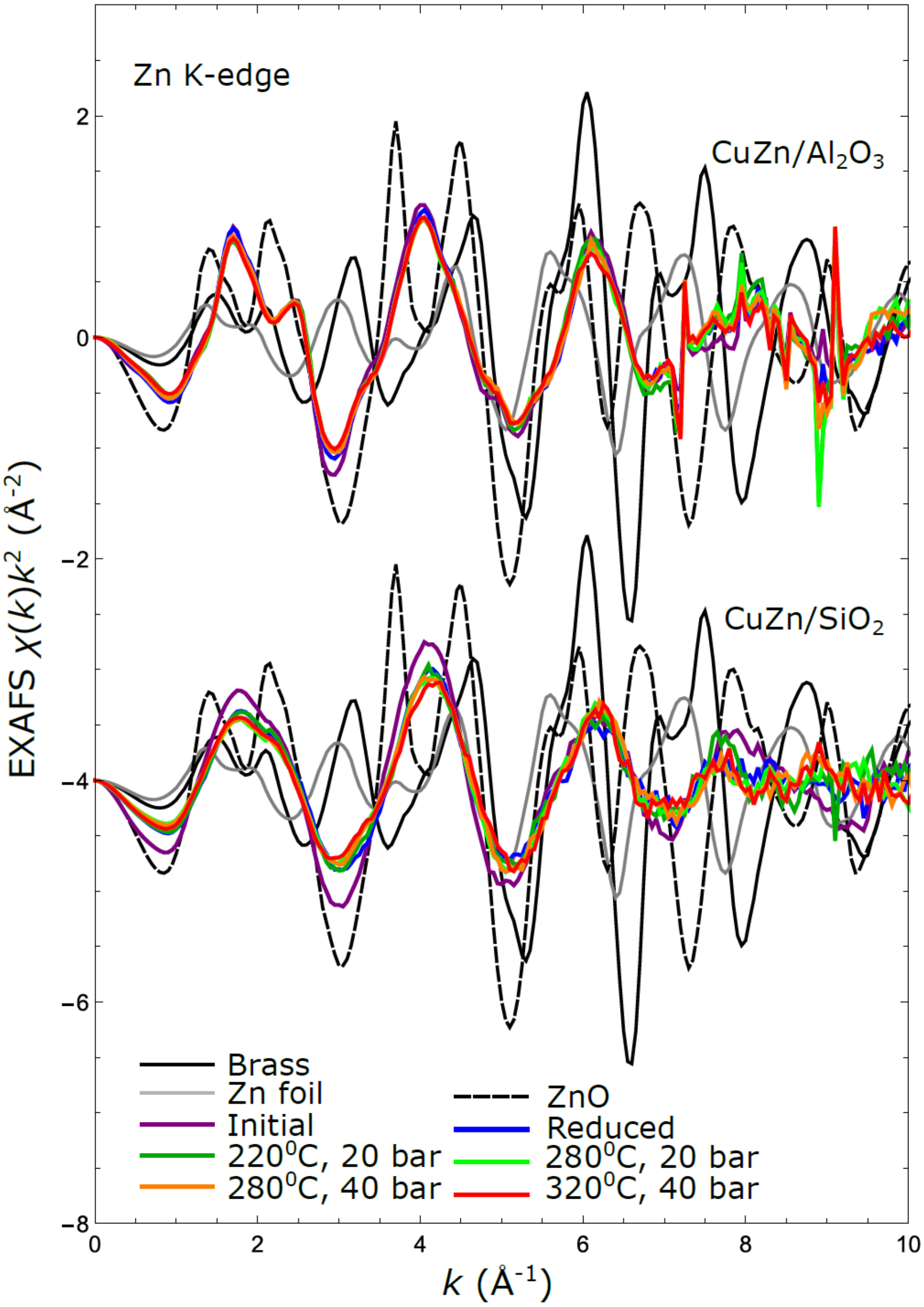

Supplementary Figure 15. *Operando* Zn K-edge EXAFS spectra ($\chi(k)k^2$) of CuZn/Al_2O_3 and CuZn/SiO_2 catalysts under CO_2 hydrogenation conditions at the different temperatures and pressures tested, as indicated on the plots. The reference spectra of a metallic Zn foil, CuZn brass foil and ZnO are also shown Spectra are shifted vertically for clarity.

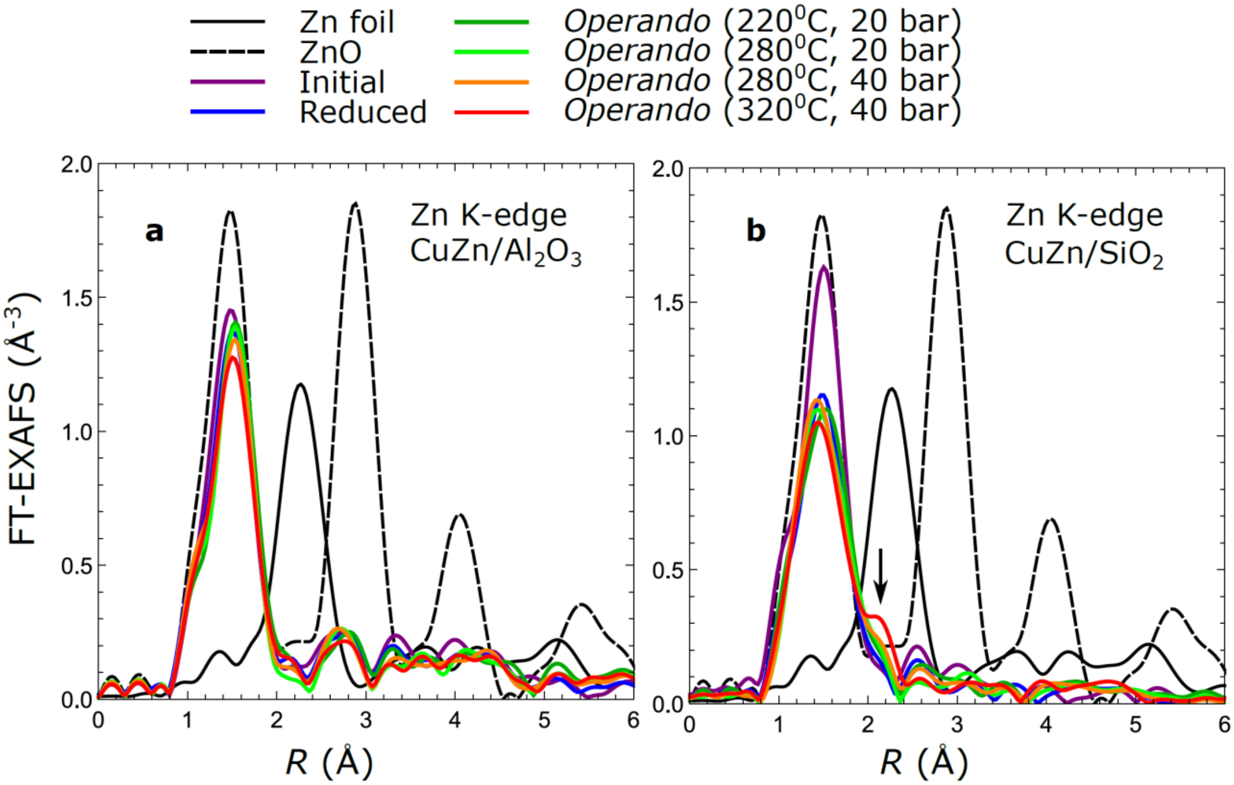

Supplementary Figure 16. Fourier-transformed *operando* Zn K-edge EXAFS spectra ($\chi(k)k^2$) of **a** CuZn/Al_2O_3 and **b** CuZn/SiO_2 catalysts under CO_2 hydrogenation conditions at different temperatures and pressures. The reference spectra of a metallic Zn foil and ZnO are also shown. The arrow marks the region where the peak corresponding to Zn—M bonds gradually develops upon catalyst activation and/or under reaction conditions.

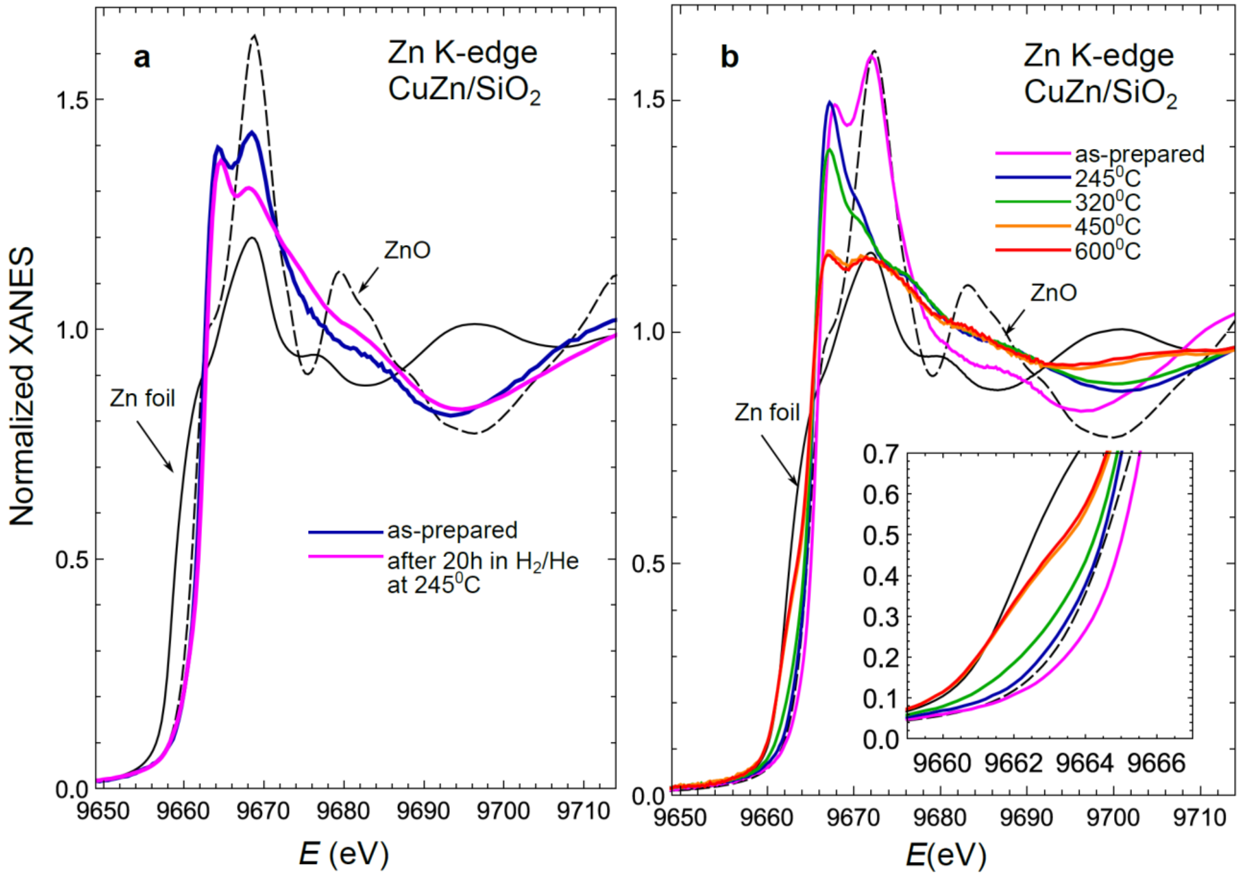

Supplementary Figure 17. Zn K-edge XANES of the $CuZn/SiO_2$ catalyst obtained in control experiments. Spectra are acquired at room temperature and 1 bar pressure for a sample in its as-prepared state, and **a** after treatment in H_2/He atmosphere at 245°C for 20 hours and **b** in a separate experiment at higher temperatures as indicated. The reference spectra of a metallic Zn foil and ZnO are also shown in both panels.

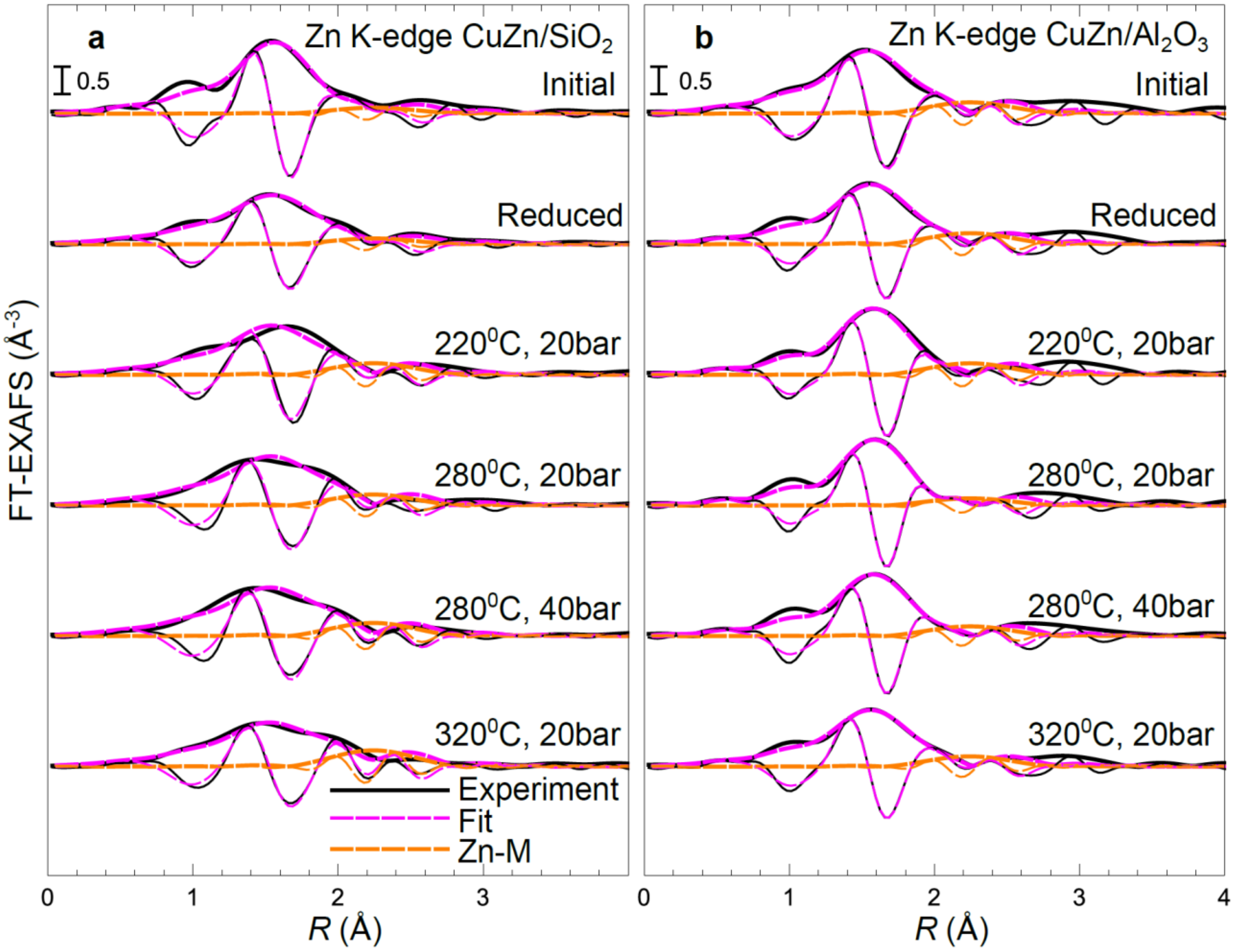

Supplementary Figure 18. Fits of Zn K-edge EXAFS spectra for **a** CuZn/SiO_2 and **b** CuZn/Al_2O_3 catalysts in their initial state at ambient conditions, during activation in hydrogen at *T*=245°C for CuZn/Al_2O_3 catalyst, *T*=325°C for CuZn/SiO_2 catalyst and under *operando* conditions at the indicated pressure and temperature. Spectra are shifted vertically for clarity. The Zn—M contribution, obtained in the EXAFS data fitting, is shown separately.

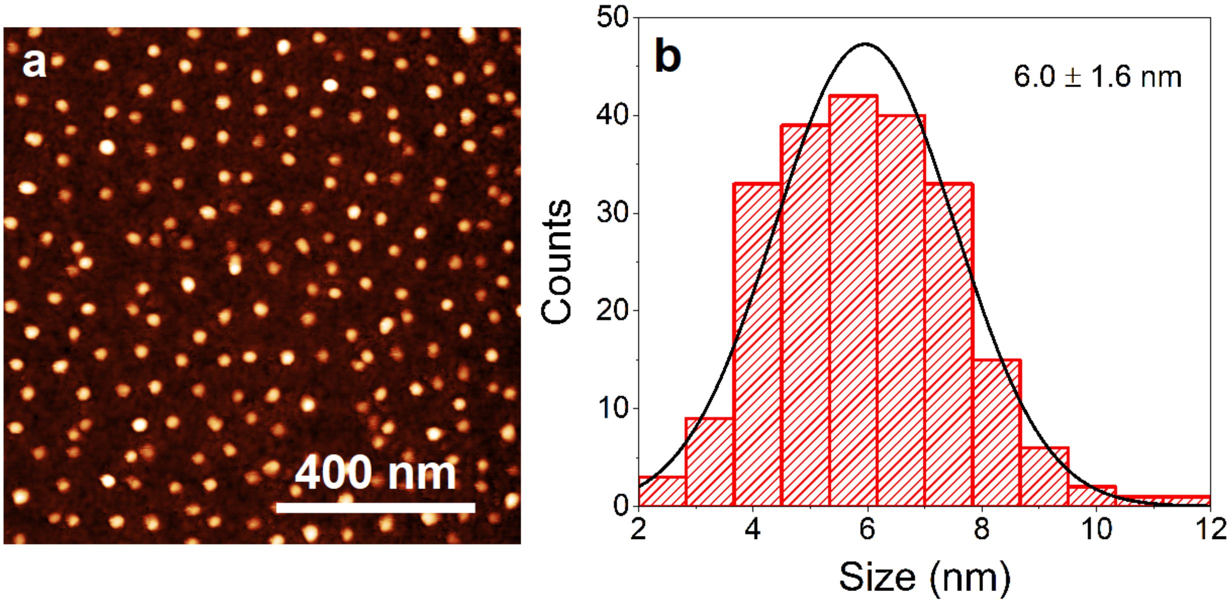

Supplementary Figure 19. a AFM image of 6 nm CuZn NPs on SiO_2/Si(100). **b** Nanoparticle height histogram obtained from the AFM image.

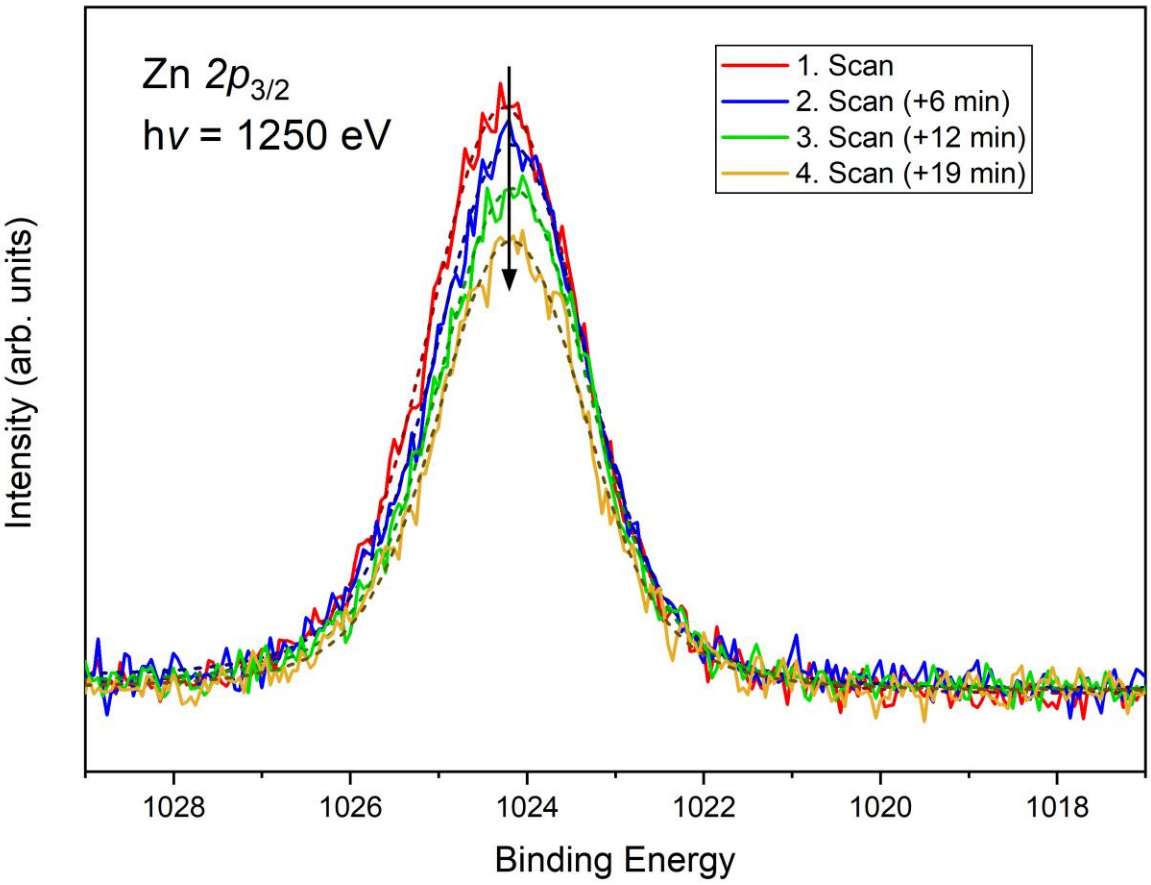

Supplementary Figure 20. Beam effect on the CuZn/SiO_2/Si(100) sample shown for consecutive Zn $2p_{3/2}$ scans in 1.3 mbar of H_2 at 350°C during ~37 min of data acquisition. The original peak area/height intensity was measured when the X-ray beam was directed to a different spot on the sample that had not been exposed to the beam before.

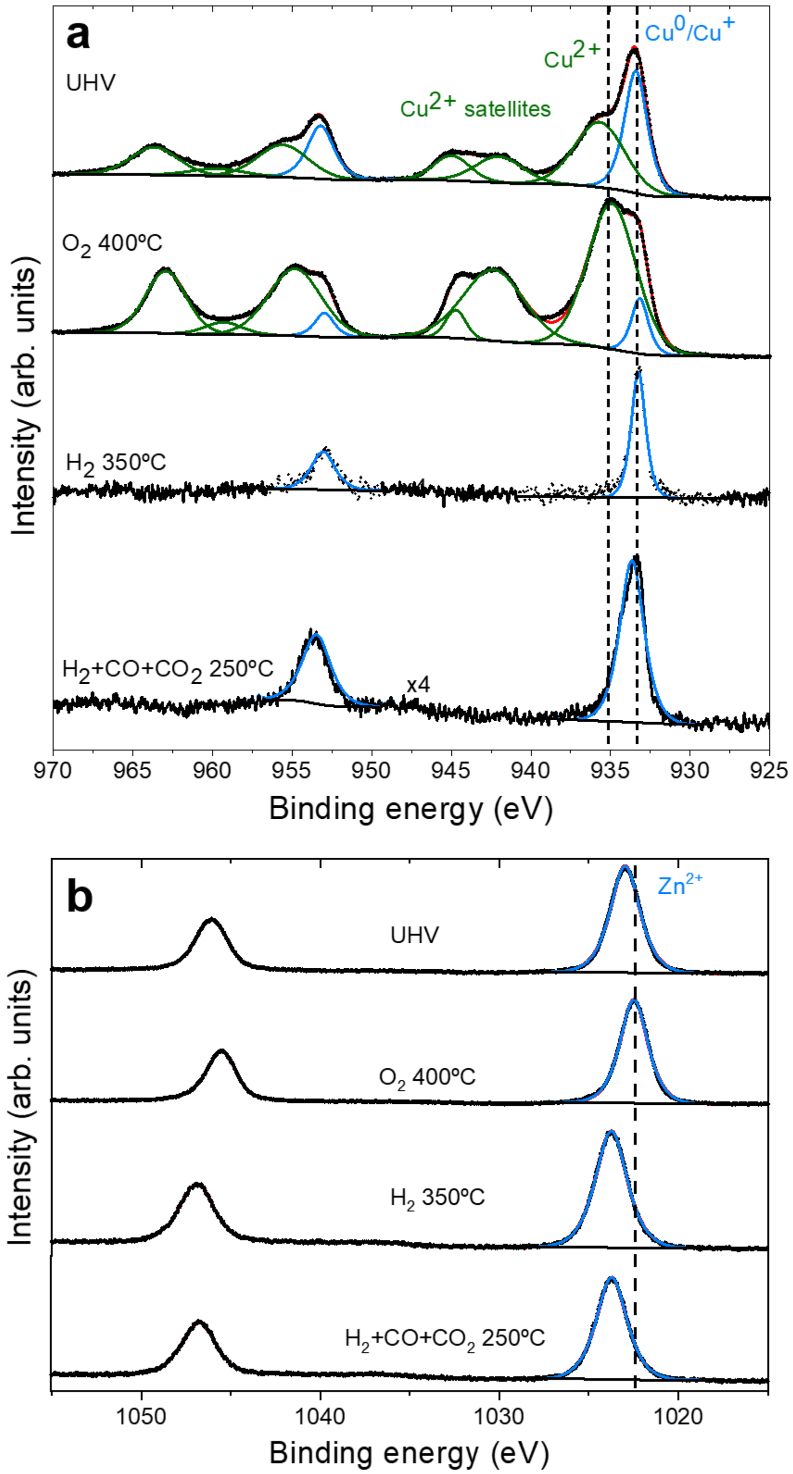

Supplementary Figure 21. Fitting of the NAP-XPS spectra of the CuZn/SiO_2/Si(100) sample for the **a** Cu *2p* and **b** Zn *2p* regions, acquired at a photon energy of 1580 eV. The fit quality is similar for the other spectra.

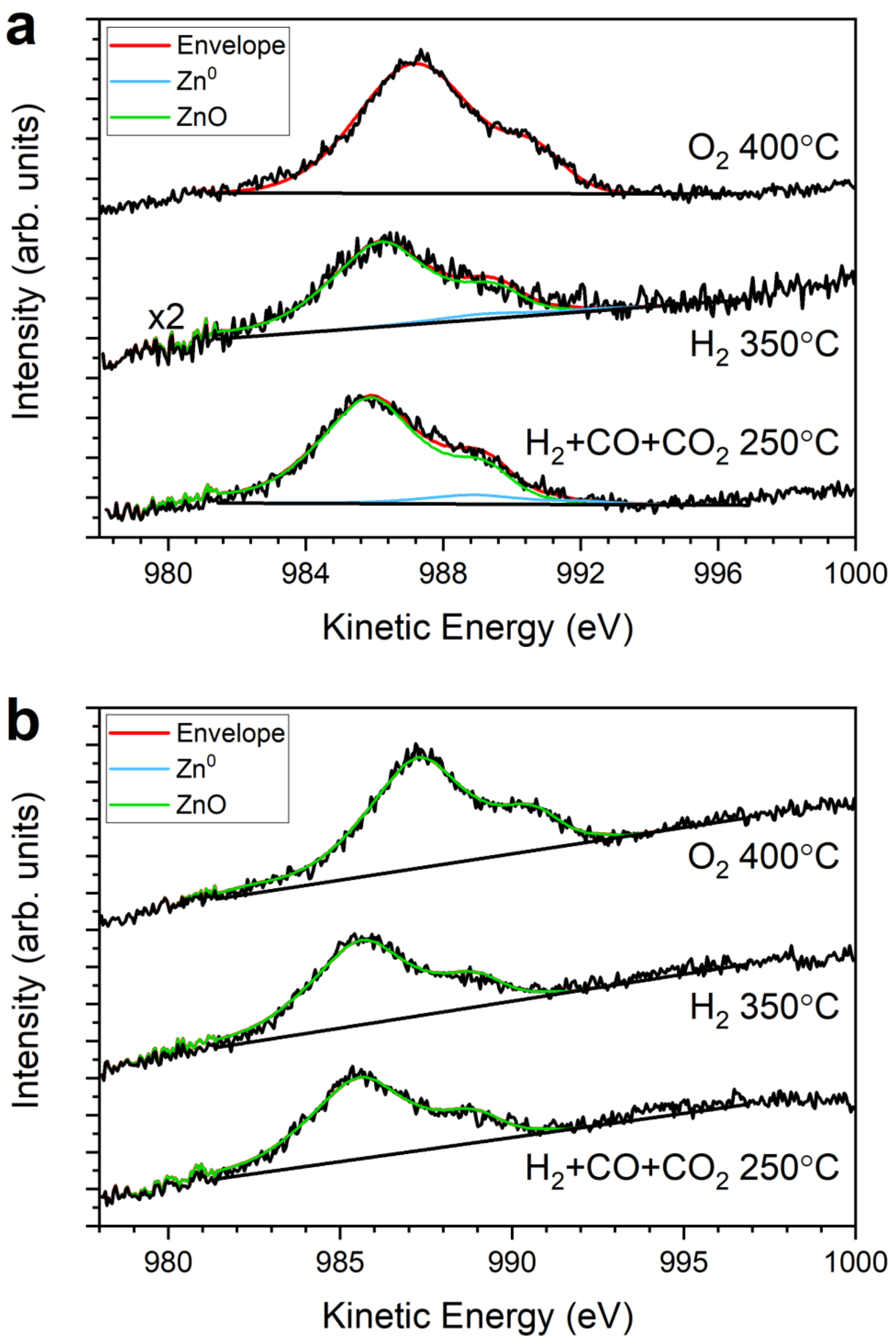

Supplementary Figure 22. NAP-XPS spectra of the CuZn/SiO_2/Si(100) sample for the Zn Auger region acquired at a photon energy of **a** 1250 eV and **b** 1580 eV for the measurement series done with the CO+CO_2+H_2 reaction gas mixture.

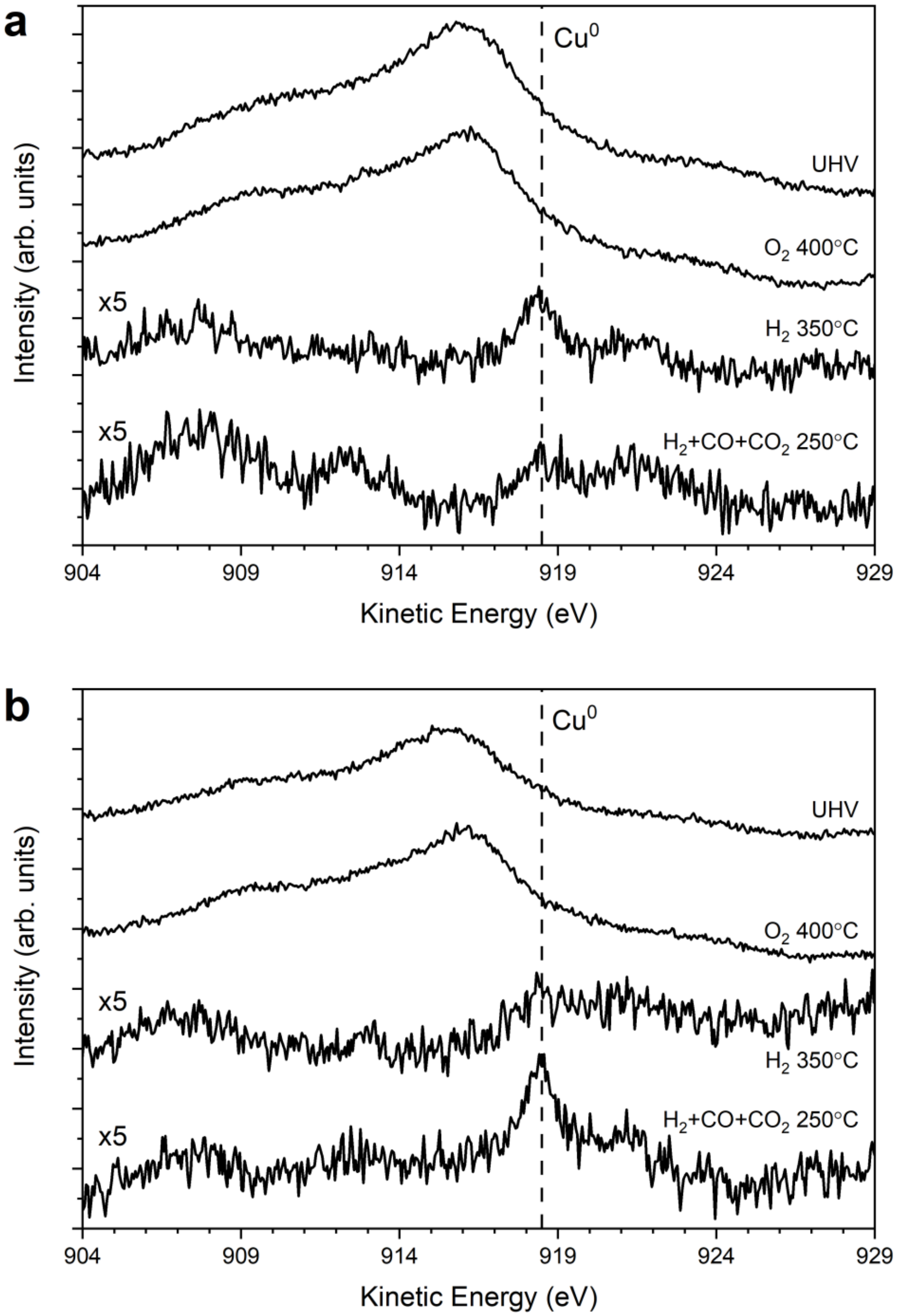

Supplementary Figure 23. NAP-XPS spectra of the $CuZn/SiO_2/Si(100)$ sample for the Cu Auger region acquired at a photon energy of **a** 1250 eV and **b** 1580 eV for the measurement series done with the $CO+CO_2+H_2$ reaction gas mixture

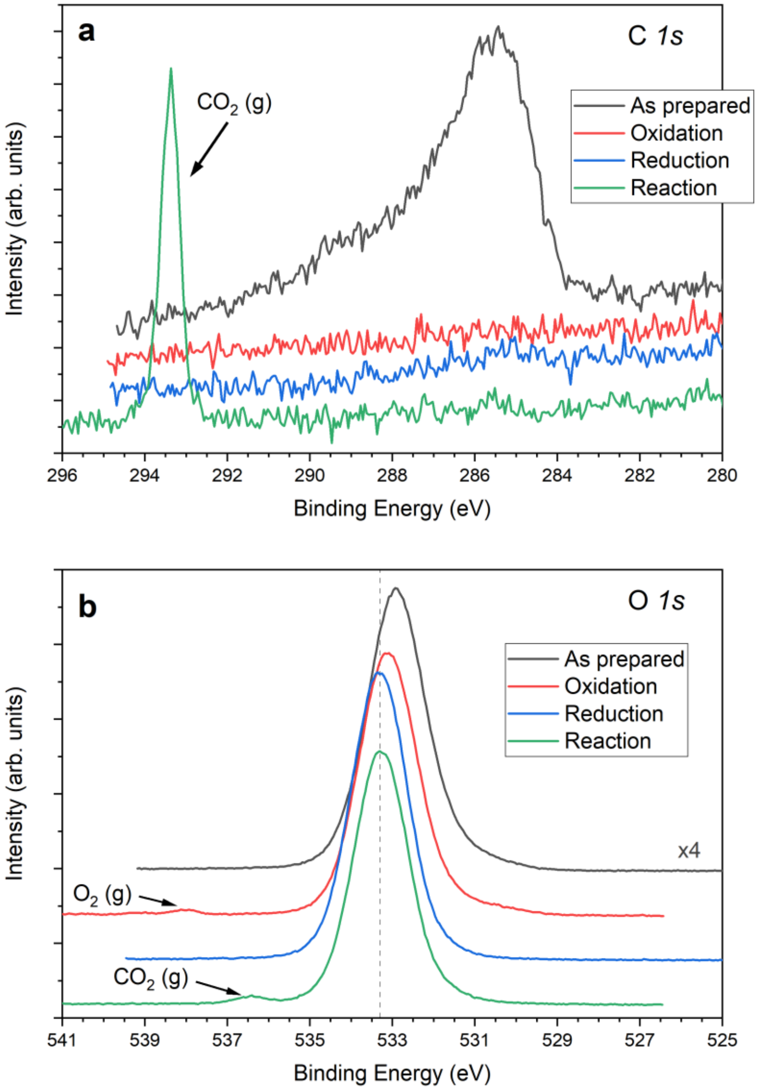

Supplementary Figure 24. **a** C *1s* and **b** O *1s* NAP-XPS spectra of CuZn NPs on SiO_2/Si(111) acquired in various gas mixtures using a photon energy of 1250 eV. **a** The as prepared spectra were measured under UHV conditions, the spectra labeled with reaction were measured in a H_2 and CO_2 containing mixture. For the C *1s* region, the peak appearing as prepared under UHV condition corresponds to adventitious carbon due to the sample transfer to the synchrotron facility in air. The adventitious C peak was completely removed during the initial oxidation pre-treatment. The peak appearing during the reaction corresponds to gas-phase CO_2. **b** For the O *1s* region, gas-phase peaks for O_2 and CO_2 are observed during oxidizing and reaction condition respectively. The main O *1s* peaks corresponds to SiO_2 from the support. No significant changes were observed for the main peak, when changing from the reducing to the reaction mixture.

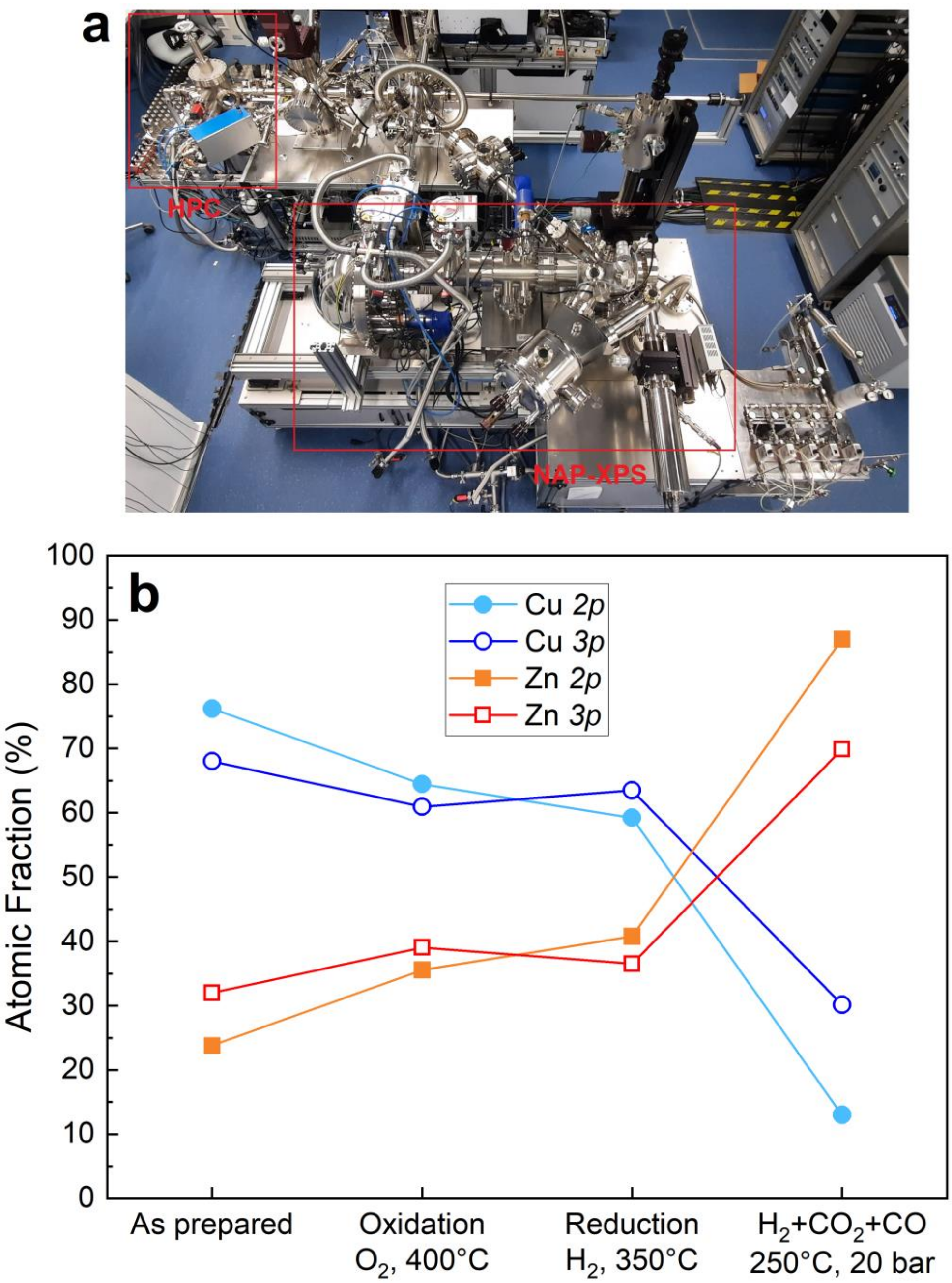

Supplementary Figure 25. a The UHV setup used for the quasi in situ experiments. The sample can be transferred from the HPC (top left), where it can be exposed to up to 20 bar of a desired gas atmosphere, to the NAP-XPS (bottom) through UHV. **b** Copper and zinc atomic fractions obtained from XPS measured acquired under UHV after exposure to different atmospheres and reaction conditions as indicated in the plot. Ratios from the *2p* and *3p* region are displayed to indicate different probing depths.

Supplementary Tables

Supplementary Table 1. Molecular weights of the polymers used in the nanoparticle synthesis. The data are given in g/mol. The metal salt loading to PVP ratio is also indicated.

Details of polymers used for nanoparticle preparation			
Catalyst	PS	P2VP	Salt/PVP ratio
CuZn/Al_2O_3	8200	8300	0.2
CuZn/SiO_2	130000	135000	0.5
Cu/ZnO/Al_2O_3	48500	70000	0.2
Cu/Al_2O_3	8200	8300	0.2
Cu/SiO_2	130000	135000	0.5

Supplementary Table 2. ICP results for the normalization of catalytic performance

ICP results			
Catalyst	Cu (%)	Zn (%)	Cu/(Cu+Zn)
CuZn/Al_2O_3	2.68(8)	1.13(4)	0.703
CuZn/SiO_2	1.39(2)	0.84(1)	0.623
Cu/ZnO/Al_2O_3	4.02(20)	-	-
Cu/Al_2O_3	4.38(14)	-	1
Cu/SiO_2	2.60(5)	-	1

Supplementary Table 3. XRD fitting parameters for $CuZn/SiO_2$ calcined

XRD fitting parameters for $CuZn/SiO_2$ calcined							
Phase	Space Group	X / wt.%	a / Å	b / Å	c / Å	β / Å	Coherence length / nm
$Cu_3Zn(OH)_6Cl_4$	R-3m	58.7(14)	6.860(6)	-	14.245(13)	-	5.6(5)
CuO	C12/c1	41.3(14)	4.686(4)	3.426(3)	5.133(4)	99.481(4)	36.2(14)

Supplementary Table 4. XRD fitting parameters for $CuZn/SiO_2$ after reaction

XRD fitting parameters for $CuZn/SiO_2$ after reaction					
Phase	Space Group	X / wt.%	a / Å	c / Å	Coherence length / nm
Cu_xZn_y	Fm-3m	72.2(15)	3.6201(5)	-	18.7(8)
Cu	Fm-3m	17.0(16)	3.6143(4)	-	85(6)
ZnO	$P6_3mc$	10.8(7)	3.2499(8)	5.202(2)	28(3)

Supplementary Table 5. XRD fitting parameters for $Cu/ZnO/Al_2O_3$ calcined

XRD fitting parameters for $Cu/ZnO/Al_2O_3$ calcined							
Phase	Space Group	X / wt.%	a / Å	b / Å	c / Å	β / Å	Coherence length / nm
ZnO	$P6_3mc$	92.3(4)	3.2516(3)	3.2516(3)	5.2076(4)	-	24(13)
CuO	C12/c1	5.7(3)	4.6876(10)	3.4299(7)	5.1336(12)	99.386(13)	22.6(7)
$Zn_5(OH)_8Cl_2{\cdot}H_2O$	R-3m	2.09(18)	6.333(4)	6.333(4)	23.62(2)	-	11.3(12)

Supplementary Table 6. XRD fitting parameters for $Cu/ZnO/Al_2O_3$ after reaction

XRD fitting parameters for $Cu/ZnO/Al_2O_3$ after reaction					
Phase	Space Group	X / wt.%	a / Å	c / Å	Coherence length / nm
ZnO	$P6_3mc$	99.21(9)	3.2510(5)	5.2074(8)	23.7(3)
Cu	Fm-3m	0.55(6)	3.6162(6)	-	74(14)
Cu_xZn_y	Fm-3m	0.24(6)	3.6260(11)	-	60(20)

Supplementary Table 7. Composition of the various gas mixtures used during the NAP-XPS measurements. The ratios were controlled by mass spectrometry.

Composition of gas mixtures used during XPS measurements				
Gas mixture	H_2 (%)	CO_2 (%)	CO (%)	H_2O (%)
$H_2 + CO_2 + CO$	84	4	12	0
$H_2 + CO_2$	75	25	0	0
$H_2 + CO_2 + H_2O$	71.4	23.8	0	4.8
$H_2 + CO_2 + CO + H_2O$	80	3.8	11.2	4

Supplementary Table 8. Best-fit parameters extracted from the analysis of Cu K-edge EXAFS spectra for the whole series of experiments and for the three catalysts investigated under *operando* conditions. Included are the coordination numbers (N), the bond lengths (R), Debye-Waller factors (σ^2) for Cu—O and Cu—M bonds, and energy shift in the photoelectron reference energy (ΔE_0). The values in parenthesis are the standard errors in the last digit. For comparison, for reduced catalysts, we also show the Cu—M coordination numbers values, extracted from XANES data using neural-network.

Best-fit parameters for CuO and catalysts in the initial state					
Sample	N_{Cu-O}	R_{Cu-O} (Å)	σ^2_{Cu-O} (Å^2)	ΔE_0 (eV)	R factor
CuO	4	1.94(1)	0.007(2)	4(2)	0.8%
Cu/ZnO/Al_2O_3 (initial)	3.9(6)	1.94(2)	0.007(3)	4(2)	0.9%
CuZn/SiO_2 (initial)	3.7(5)	1.95(2)	0.007(2)	4(2)	0.8%

Best-fit parameters for catalysts during and after the activation treatment								
Sample	N_{Cu-O}	R_{Cu-O} (Å)	σ^2_{Cu-O} (Å^2)	N_{Cu-M}	R_{Cu-M} (Å)	σ^2_{Cu-M} (Å^2)	ΔE_0 (eV)	R factor
Cu/ZnO/Al_2O_3 (activation, 245°C)	-	-	-	8.3(7)	2.522(6)	0.014(1)	0.0(7)	0.4%
CuZn/Al_2O_3 (activation, 245°C)	0.6(7)	1.94(5)	0.02(2)	5.5(9)	2.527(9)	0.012(1)	1(1)	0.5%
CuZn/SiO_2 (activation, 325°C)	-	-	-	8.9(9)	2.519(7)	0.017(1)	-0.4(9)	0.7%
CuZn/SiO_2, 25°C, 1 bar, after activation at 245°C for 2h	1(2)	1.98(9)	0.02(5)	9.9(7)	2.57(2)	0.009(1)	-8(2)	0.2%
CuZn/SiO_2, 25°C, 1 bar, after activation at 245°C for 20h	-	-	-	10.3(8)	2.540(5)	0.009(1)	-10.3(9)	0.8%

Best-fit parameters for catalysts under reaction conditions and after reaction						
Sample	N_{Cu-M} ($XANES$)	N_{Cu-M} ($EXAFS$)	R_{Cu-M} (Å)	σ^2_{Cu-M} (Å^2)	ΔE_0 (eV)	R factor
Cu foil	12.1(2)	12	2.541(4)	0.009(1)	2.5(6)	0.2%
Cu/ZnO/Al_2O_3 , 220°C, 20 bar	10.1(6)	9.2(4)	2.526(3)	0.012(1)	0.5(5)	0.5%
Cu/ZnO/Al_2O_3 , 280°C, 20 bar	9.9(6)	8.3(7)	2.524(6)	0.012(1)	0.2(8)	0.1%
Cu/ZnO/Al_2O_3 , 280°C, 40 bar	10.1(7)	8.9(5)	2.522(4)	0.013(1)	0.0(5)	0.5%
Cu/ZnO/Al_2O_3 , 320°C, 40 bar	9.9(7)	9.6(8)	2.528(6)	0.016(1)	0.3(8)	0.5%
CuZn/Al_2O_3, 220°C, 20 bar	8.5(8)	7.6(7)	2.535(6)	0.012(1)	1.8(8)	0.5%
CuZn/Al_2O_3, 280°C, 20 bar	8.4(8)	7.5(6)	2.529(6)	0.013(1)	1.3(8)	0.5%
CuZn/Al_2O_3, 280°C, 40 bar	8.5(9)	7.2(5)	2.528(4)	0.013(1)	1.2(6)	0.3%
CuZn/Al_2O_3, 320°C, 40 bar	8.3(8)	7.3(6)	2.527(5)	0.014(1)	0.9(7)	0.4%
CuZn/SiO_2, 220°C, 20 bar	10.1(7)	10(1)	2.523(7)	0.013(1)	0(1)	0.7%
CuZn/SiO_2, 280°C, 20 bar	9.9(7)	10.1(7)	2.535(5)	0.015(1)	1.0(6)	0.3%
CuZn/SiO_2, 280°C, 40 bar	9.9(7)	9.8(7)	2.531(5)	0.015(1)	0.4(7)	0.4%
CuZn/SiO_2, 320°C, 40 bar	9.6(8)	10(1)	2.532(7)	0.017(1)	0.4(8)	0.6%
CuZn/SiO_2, 25°C, 1 bar, after reaction at 250°C/20 bar	-	9.7(8)	2.543(5)	0.008(1)	-10.4(8)	0.7%

Supplementary Table 9. Best-fit parameters extracted from the analysis of Zn K-edge EXAFS spectra for the CuZn/Al_2O_3 and CuZn/SiO_2 catalysts. Included are the coordination numbers (N), the bond lengths (R), EXAFS Debye-Waller factors (σ^2), and photoelectron reference energy shift, ΔE_0. The values in parenthesis are the standard errors in the last digit.

Best-fit parameters for ZnO								
Sample	N_{Zn-O}	R_{Zn-O} (Å)	σ^2_{Zn-O} (Å^2)	N_{Zn-M}	R_{Zn-M} (Å)	σ^2_{Zn-M} (Å^2)	ΔE_0 (eV)	R factor
ZnO	4	1.970(4)	0.0053(6)	-	-	-	-2.2(5)	0.07%
Best-fit parameters for CuZn/SiO_2								
Sample	N_{Zn-O}	R_{Zn-O} (Å)	σ^2_{Zn-O} (Å^2)	N_{Zn-M}	R_{Zn-M} (Å)	σ^2_{Zn-M} (Å^2)	ΔE_0 (eV)	R factor
CuZn/SiO_2 initial	3.7(3)	2.01(1)	0.006(1)	0.1(1)	2.56(1)	0.003(5)	0.6(7)	2.3%
CuZn/SiO_2 (activation, 325℃)	3.3(6)	2.01(1)	0.010(3)	0.1(2)	2.56(1)	0.011(5)	2(1)	1.4%
CuZn/SiO_2, 220℃, 20 bar	3.2(8)	2.01(1)	0.009(4)	0.3(3)	2.56(1)	0.009(5)	1(1)	3.4%
CuZn/SiO_2, 280℃, 20 bar	3.3(4)	2.01(1)	0.010(2)	0.3(2)	2.56(1)	0.010(5)	1.2(8)	3.2%
CuZn/SiO_2, 280℃, 20 bar	3.5(6)	2.01(1)	0.011(3)	0.3(2)	2.56(1)	0.010(5)	1.3(9)	14.3%
CuZn/SiO_2, 320℃, 40 bar	3.2(7)	2.01(1)	0.011(4)	0.4(3)	2.56(1)	0.011(5)	2(1)	3.0%
Best-fit parameters for CuZn/Al_2O_3								
Sample	N_{Zn-O}	R_{Zn-O} (Å)	σ^2_{Zn-O} (Å^2)	N_{Zn-M}	R_{Zn-M} (Å)	σ^2_{Zn-M} (Å^2)	ΔE_0 (eV)	R factor
CuZn/Al_2O_3 initial	4(1)	2.01(1)	0.007(6)	0.3(4)	2.56(1)	0.003(5)	0(2)	2.7%
CuZn/Al_2O_3 (activation, 245℃)	3(1)	2.01(1)	0.005(6)	0.2(3)	2.56(1)	0.009(5)	1(2)	2.0%
CuZn/Al_2O_3, 220℃, 20 bar	2(1)	2.01(1)	0.001(6)	0.3(4)	2.56(1)	0.008(5)	1(3)	2.2%
CuZn/Al_2O_3, 280℃, 20 bar	2(1)	2.01(1)	0.001(7)	0.2(4)	2.56(1)	0.010(5)	2(3)	2.8%
CuZn/Al_2O_3, 280℃, 20 bar	3(1)	2.01(1)	0.003(7)	0.2(4)	2.56(1)	0.010(5)	2(3)	2.7%
CuZn/Al_2O_3, 320℃, 40 bar	3(1)	2.01(1)	0.005(8)	0.2(3)	2.56(1)	0.011(5)	1(3)	1.5%
Best-fit parameters for CuZn/SiO_2 at room temperature and 1 bar								
Sample	N_{Zn-O}	R_{Zn-O} (Å)	σ^2_{Zn-O} (Å^2)	N_{Zn-M}	R_{Zn-M} (Å)	σ^2_{Zn-M} (Å^2)	ΔE_0 (eV)	R factor
CuZn/SiO_2, 25℃, 1 bar, after activation at 245℃ for 2h	3.9(7)	1.99(2)	0.009(3)	0.1(3)	2.56(2)	0.006(5)	2(2)	0.2%
CuZn/SiO_2, 25℃, 1 bar, after reaction at 250℃/20 bar	3.8(5)	1.98(1)	0.009(2)	0.3(3)	2.56(2)	0.006(5)	1(2)	0.4%
CuZn/SiO_2, 25, 1 bar, after activation at 245℃ for 20h	3.9(2)	1.96(1)	0.008(1)	0.4(3)	2.56(2)	0.006(5)	0(1)	1.2%

Supplementary References

1. Newville, M. *et al.* Analysis of multiple-scattering XAFS data using theoretical standards. *Phys. B Phys. Condens. Matter* **208–209**, 154–156 (1995).

2. Ankudinov, A. & Ravel, B. Real-space multiple-scattering calculation and interpretation of x-ray-absorption near-edge structure. *Phys. Rev. B - Condens. Matter Mater. Phys.* **58**, 7565–7576 (1998).

3. Kuzmin, A. *et al.* Effect of Pressure and Temperature on the Local Structure and Lattice Dynamics of Copper(II) Oxide. in *Physics Procedia* vol. 85 27–35 (2016).

4. Timoshenko, J. *et al.* Subnanometer Substructures in Nanoassemblies Formed from Clusters under a Reactive Atmosphere Revealed Using Machine Learning. *J. Phys. Chem. C* **122**, 21686–21693 (2018).

5. Grunwaldt, J. D., Molenbroek, A. M., Topsøe, N. Y., Topsøe, H. & Clausen, B. S. In situ investigations of structural changes in Cu/ZnO catalysts. *J. Catal.* **194**, 452–460 (2000).

6. Timoshenko, J. & Frenkel, A. I. Probing structural relaxation in nanosized catalysts by combining EXAFS and reverse Monte Carlo methods. *Catal. Today* **280**, 274–282 (2017).

7. Biesinger, M. C., Lau, L. W. M., Gerson, A. R. & Smart, R. S. C. Resolving surface chemical states in XPS analysis of first row transition metals, oxides and hydroxides: Sc, Ti, V, Cu and Zn. *Appl. Surf. Sci.* **257**, 887–898 (2010).
